# Pyrosequencing Reveals a Core Community of Anodic Bacterial Biofilms in Bioelectrochemical Systems from China

**DOI:** 10.3389/fmicb.2015.01410

**Published:** 2015-12-16

**Authors:** Yong Xiao, Yue Zheng, Song Wu, En-Hua Zhang, Zheng Chen, Peng Liang, Xia Huang, Zhao-Hui Yang, I-Son Ng, Bor-Yann Chen, Feng Zhao

**Affiliations:** ^1^Key Laboratory of Urban Pollutant Conversion, Institute of Urban Environment, Chinese Academy of SciencesXiamen, China; ^2^College of Environmental Science and Engineering, Hunan UniversityChangsha, China; ^3^Research Center for Eco-Environmental Sciences, Chinese Academy of SciencesBeijing, China; ^4^School of Environment, Tsinghua UniversityBeijing, China; ^5^Department of Chemical Engineering, National Cheng Kung UniversityTainan, Taiwan; ^6^Department of Chemical and Materials Engineering, National I-Lan UniversityI-Lan, Taiwan

**Keywords:** high-throughput sequencing, microbial community, electrochemically active microorganisms, microbial fuel cells, bioelectrochemical systems, electron transfer

## Abstract

Bioelectrochemical systems (BESs) are promising technologies for energy and product recovery coupled with wastewater treatment, and the core microbial community in electrochemically active biofilm in BESs remains controversy. In the present study, 7 anodic communities from 6 bioelectrochemical systems in 4 labs in southeast, north and south-central of China are explored by 454 pyrosequencing. A total of 251,225 effective sequences are obtained for 7 electrochemically active biofilm samples at 3% cutoff level. While Alpha-, Beta-, and Gamma-proteobacteria are the most abundant classes (averaging 16.0–17.7%), Bacteroidia and Clostridia are the two sub-dominant and commonly shared classes. Six commonly shared genera i.e., *Azospira, Azospirillum, Acinetobacter, Bacteroides, Geobacter, Pseudomonas*, and *Rhodopseudomonas* dominate the electrochemically active communities and are defined as core genera. A total of 25 OTUs with average relative abundance >0.5% were selected and designated as core OTUs, and some species relating to these OTUs have been reported electrochemically active. Furthermore, cyclic voltammetry and chronoamperometry tests show that two strains from *Acinetobacter guillouiae* and *Stappia indica*, bacteria relate to two core OTUs, are electrochemically active. Using randomly selected bioelectrochemical systems, the study has presented extremely diverse bacterial communities in anodic biofilms, though, we still can suggest some potentially microbes for investigating the electrochemical mechanisms in bioelectrochemical systems.

## Introduction

Bioelectrochemical systems (BESs) are promising technologies for energy and products recovery coupled with wastewater treatment and have attracted increasing attention (Liu and Logan, 2004; Lovley, 2006a; Zhao et al., 2009b; Liu et al., 2013). Many studies have been conducted to expand the application of BESs and increase the efficiency of electricity production (Zhao et al., 2009a; Zhou et al., 2011; Xiao et al., 2013a). Microorganisms are believed to play key roles in electricity production (Logan and Regan, 2006; Lovley, 2006b), and extracellular electron transfer, the basis of electricity production, is the most frequent concern in BESs research. However, most studies on extracellular electron transfer are focusing on the two model species of *Shewanella oneidensis* (Marsili et al., 2008; Jiang et al., 2010) and *Geobacter sulfurreducens* (Reguera et al., 2005; Shrestha et al., 2013).

Electrochemically active microorganisms (EAMs) are a group of microorganisms which can transfer electrons from cells to an electron acceptor or accept electrons from an electron donor. Up to date, several EAMs have been identified (Xiao et al., 2013b). However, the understanding of performance of electrochemically active biofilm (EAB), which consists of EAMs and other microorganisms, is still poor due to limited knowledge on the microbial community in EAB.

Similar to soil and activated sludge, EAB is a very complex system consisting of viruses, bacteria, archaea, and fungi. However, the microbial community in EAB remains largely unstudied. This should be partly ascribed to the lack of robust techniques required to explore the highly complex community. In previous studies on the microbial community in EAB, denaturing gradient gel electrophoresis (DGGE) and cloning library are two commonly used techniques (Jung and Regan, 2007; Sun et al., 2011; Beecroft et al., 2012; Liang et al., 2013), and these traditional molecular approaches provide relative low sequencing depth while compared with the vast microbial diversity in EAB. The current investigations merely represent a snapshot of some dominant species in EAB community without providing information on species with medium to low abundances.

High-throughput sequencing, which can provide enough sequencing depth to cover a complex microbial community (Shendure and Ji, 2008), is a promising technology to explore the microbial communities in EAB. Up to now, the method has been widely used to analyze environmental microbial communities in marine water (Stoeck et al., 2010), activated sludge (Zhang et al., 2012), soil (Rousk et al., 2010), and also in BESs (Lee et al., 2010; Miceli et al., 2012; Xiao et al., 2015), these studies gained limited sequences. Therefore, people have not yet pictured full profiles of the microbial communities in EAB.

Six anodic EAB samples were randomly collected from four laboratories in Beijing, Changsha, and Xiamen in China. The 454 pyrosequencing targeting 16S rRNA genes was used (1) to profile the abundance, diversity, and composition of different anodic communities, (2) to investigate whether there are commonly shared species in randomly selected anodic EAB, (3) to compare the variability in anodic EAB during substrate replacing, and (4) to confirm whether dominant species in EAB are electrochemically active.

## Experimental

### Samples

The samples of anodic biofilm were collected from six BESs at steady operation in Changsha (CS-LXM) (Liu et al., 2009), Beijing (BJ-CZ, BJ-HX) and Xiamen (XM1, XM2, and XM3). As the object of the study was to profile the bacterial communities and summarize the core members in anodic biofilm, we only showed some common information of the stably operated BESs (Table 1). To test the reproducibility of pyrosequencing, sample BJ-HX was divided into two samples (designated as BJ-HX1 and BJ-HX2) and subjected to sequential DNA extraction, PCR and pyrosequencing.

**Table 1:** **Characteristics of the anodic electrochemically active biofilm samples**.

**Code**	**Source (name, city, and location)**	**Substrate**	**Inoculant**	**Electrode material**	**Output power density/W m^−3^**
CS-LXM	Prof. Xiao-Ming Li, Changsha, central south China	Excess sludge	Activated sludge	Carbon cloth	0.31 (Liu et al., 2009)
BJ-CZ	Dr. Zheng Chen, Beijing, north China	Sodium acetate	Paddy soil	Carbon cloth	12.5
BJ-HX1 and 2	Prof. Xia Huang, Beijing, north China	Sodium acetate	Activated sludge	Carbon granucle	38.2
XM1^a^	Dr. Yong Xiao, Xiamen, southeast China	Sodium acetate	Activated sludge	Carbon felt	10.1
XM2^a^		Organic kitchen waste	—	Carbon felt	9.5
XM3^a^		Sodium acetate	—	Carbon felt	9.7

a *The three samples were collected from the same BES which was sequentially fed with sodium acetate, organic kitchen waste, and sodium acetate. Each material was used as substrate for 60 days*.

### DNA extraction

For each sample, the genomic DNA was extracted by a previously reported protocol using CTAB and proteinase K (Yang et al., 2007), which can successfully extracted genomic DNA from microbes in various environments e.g., compost and sludge (Xiao e al., 2009; Xiao et al., 2011a,b). The extracted genomic DNA was purified with a kit (DP1501, BioTeke, China). DNA quality was assessed by agarose gel electrophoresis and the 260/280- and 260/230-nm absorption ratios on an ND-2000 spectrophotometer (Nanodrop, USA).

### PCR and 454 pyrosequencing

Before pyrosequencing, the purified DNA was amplified with a set of primers targeting the V1-V3 hypervariable regions of bacterial 16S rRNA genes. The forward primer was 5′-AGAGTTTGATCCTGGCTCAG-3′ (27F) with the Roche 454 “B” adapter, and the reverse primer was 5′- TTACCGCGGCTGCTGGCAC -3′ (533R) which containing the Roche 454 “A” adapter and specific 10 bp barcode. The Roche 454 “A”/“B” adapter located on the 5′-end of each primer, respectively. Each 20 μL of PCR reaction system contained 4 μL of 5 × FastPfu buffer, 2 μL of dNTPs (2.5 mM), 0.8 μL of forward primer (5 μM), 0.8 μL of reverse primer (5 μM), 0.4 μL of FastPfu polymerase and 10 ng of template DNA (the rest of bulk was Milli-Q water). The PCR amplification followed the conditions: one cycle of initial denaturation at 95°C for 2 min; 25 cycles of denaturation at 95°C for 30 s, annealing at 55°C for 30 s and extension at 72°C for 30 s; a final extension at 72°C for 5 min.

The PCR products were quantitated by QuantiFluor™ -ST (Scientific Products, USA) and then mixed for pyrosequencing. The high-throughput pyrosequencing was processed on Roche GS FLX+ System (Roche, USA).

### Post-run analysis

The pyrosequencing data were processed using Quantitative Insights Into Microbial Ecology (QIIME) pipeline (Caporaso et al., 2010b). Before the statistical analysis of data, QIIME were used to (1) check the completeness of the barcodes and the primer sequencing, (2) remove reads shorter than 200 bp, and (3) remove reads comprising chimera and quality score below 25. Secondly, the sequences belonging to different samples were exactly assigned using the unique 10 bp barcodes from raw data, and then the barcodes sequences were removed. Only the 97% identity of the effective sequences were divided into OTUs for further analysis, and the most abundant sequence from each OTU was selected as the representative sequence by PyNAST (Caporaso et al., 2010a). Then, these representative sequences were used for the classification of taxonomic according the Greengenes database. The sequences were used to explore the Alpha-diversity in each sample, and the UniFrac metric was employed to compare the beta-diversity between samples. To study the diversity in every sample, the number of OTUs, Chao1 index and phylogenetic richness index diversity were calculated from each sample. In the light of OTUs table, we performed principal component analysis by R v.2.15.0. OTU abundance is relative abundance determined by dividing the sequence number of any given OTU by the total sequence number of that sample.

### Electrochemical test

The strain of *Acinetobacter guillouiae* Ax-9 was screened from Yi-lan in Taiwan (Ng et al., 2014) and cultured in LB broth at 37°C. The strain of *Stappia indica* MCCC 1A01226 was bought from Marine Culture Collection of China (Lai et al., 2010) and cultured with Difco™ Marine Broth 2216 (BD, USA) at 25°C. The cyclic voltammetry (CV) test was anaerobically conducted on glassy carbon electrode using strains at the late-log growth phase after washed twice with PBS buffer (pH 7.0) (Wu et al., 2014). Chronoamperometry (CA) measurements were conducted in three-electrode systems where carbon felts (9 cm; Liu et al., 2013), stainless steel mesh (9 cm^2^) and saturated Ag/AgCl were used as working electrode, counter electrode and reference electrode, respectively. LB broth and Difco™ Marine Broth 2216 were added as culturing medium, respectively, and the reactor was sealed by rubber seal to maintain anaerobic condition in the chamber. During the test, 1% of bacteria suspension was inoculated into the reactors and a potential of +0.30 V (vs. Ag/AgCl) was applied onto the working electrodes by an electrochemistry workstation. In the abiotic control experiment, only LB broth or Difco™ Marine Broth 2216 was added into the reactor.

### Nucleotide sequence accession numbers

Twenty-five OTU sequences with high average abundance in the 7 samples have been deposited in the GenBank database under accession numbers KJ009261-KJ009285.

## Results and discussion

### Overall pyrosequencing information

After filtering the low quality reads (length <200 bp and quality score <25) and trimming adapters and barcodes, a total of 266,791 high quality reads were obtained for the 7 EAB samples using QIIME (Table S1). Then, the high quality reads were denoised, and chimeras were filtered out. There were 30,269–43,868 effective reads (a total of 251,225 sequences) for the 7 EAB samples. Since the smallest library among the 7 samples consisted of 30,269 sequences, the library size of each EAB sample was normalized to 30,269 sequences, and the downstream analyses for different samples were conducted at the same sequencing depth of 30,269 sequences. For the selected 211,883 sequences (7 samples), the average length of sequences is 485.8 bp. According to the Pearson correlation coefficient for the relation between samples BJ-HX1 and BJ-HX2 (*R*^2^ = 0.903; Figure S1), the pyrosequencing showed high repeatability and reliability.

### Diversity of bacterial communities

The numbers of operational taxonomic units (OTUs), Chao 1, Phylogenetic Diversity index and Good's coverage at the cutoff level of 3% are summarized in Table S1. Good's coverage shows the sampling completeness and the probability i.e., the possibility of a randomly selected sequence from the sequence data of a sample has already been sequenced. Though, 30,269 sequences for each sample were obtained from pyrosequencing, the Good's coverage ranged between 78.4 and 98.2%. The Good's coverage result together with the plots of OTU number, Chao 1 and Phylogenetic Diversity Index vs. sequence number (Figure S2) demonstrated that enough sequence depth was necessary for profiling complex EAB samples.

Based on the 251,225 effective sequences, a total of 31,502 OTUs were detected in 7 EAB samples. While each OTU could be assigned to a different microbial species, the diversity in EAB community is considerably rich, even higher than that in activated sludge (Zhang et al., 2012). There were only 1129 OTUs in sample BJ-CZ, while the OTU numbers in the other 6 samples varied from 3695 to 9468 and were 3.5–8.5 times of that in this sample. According to the three indices of OTUs, Chao 1 and Phylogenetic Diversity index, the lowest diversity of community was found in sample BJ-CZ which was inoculated with paddy soil. The other 6 BESs were inoculated with activated sludge. Some studies, at the same sequencing depth, show that the bacterial diversity in soil is as rich as or even richer than that in activated sludge (Zhang et al., 2012). However, the results in this study seemed to demonstrate that BESs inoculated with activated sludge had considerably richer community diversity than that inoculated with soil. Some studies, at the same sequencing depth, show that the bacterial diversity in soil is as rich as or even richer than that in activated sludge (Zhang et al., 2012). However, the results in this study seemed to demonstrate that BESs inoculated with activated sludge had considerably richer community diversity than that inoculated with soil. The microbial diversity in CS-LXM and XM3 was considerably richer than that in the other samples, which might be attributable to the complex organic substrates of excess sludge and organic kitchen waste in these BESs.

**Figure 1:**
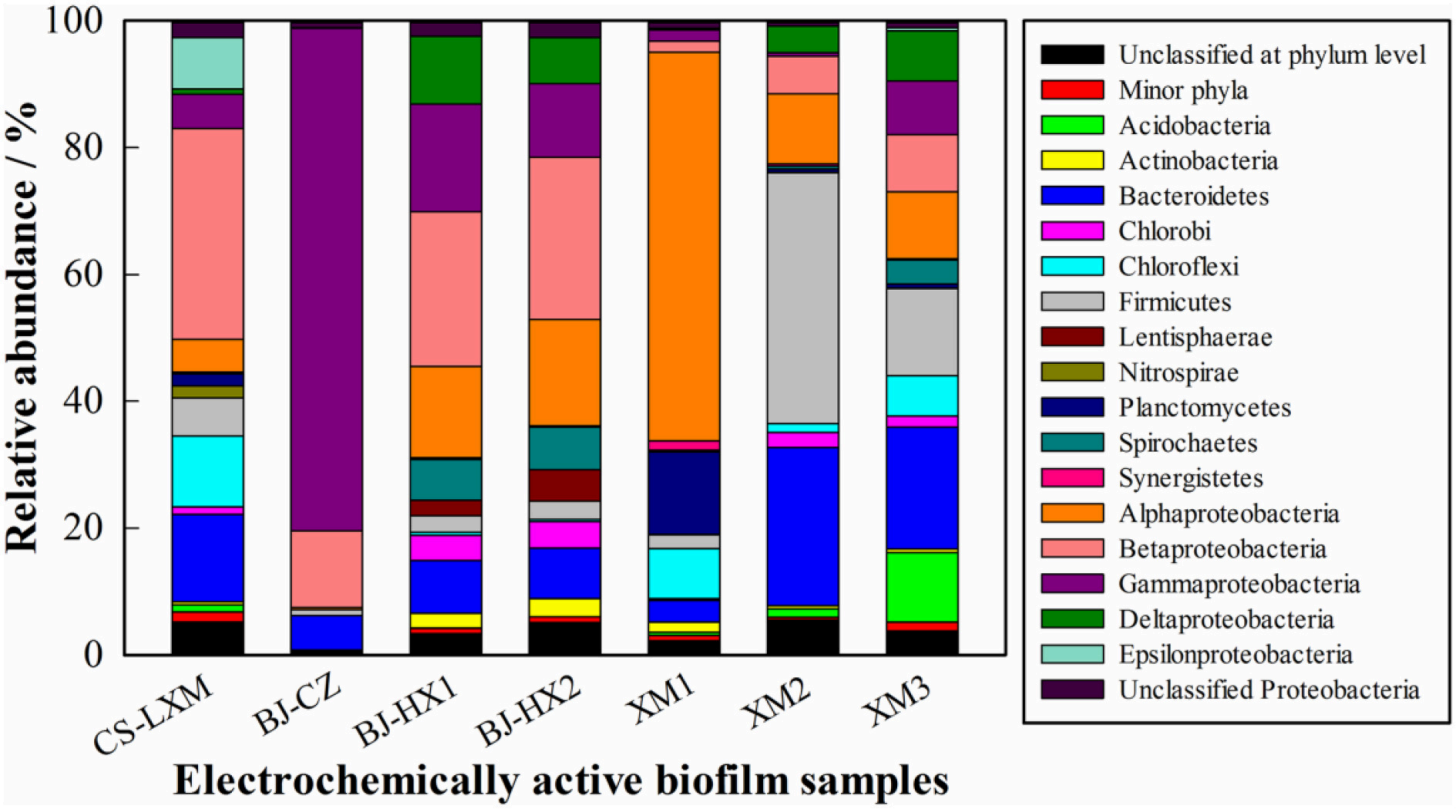
**Relative abundances of major phyla (>1% in at least one sample) and classes in Proteobacteria in the 7 EAB samples**. The relative abundance is presented as the percentage in 30269 effective sequences in each sample. Minor phyla refer to taxa with a maximum relative abundance <1% in any sample.

In most of samples (6 in 7), Proteobacteria was the most dominant phylum (Figure 1), accounting for 37.5–92.8% of the 211,883 sequences (30,269 sequences for each sample). The results were similar to previous reports on anodic communities (Lee et al., 2010; Yates et al., 2012) or isolated EAMs (Xiao et al., 2013b). In these 6 samples, Bacteroidetes (3.4–19.2%, averaging at 9.7%), Firmicutes (0.9–13.8%, averaging at 4.7%), and Chloroflexi (0.02–11.2%, averaging at 4.4%) were the other sub-dominant phyla. Unlike that in the other 6 samples and previous reports (Lee et al., 2010; Miceli et al., 2012; Yates et al., 2012), Firmicutes was the most (39.6%) abundant phylum in sample XM2, while Bacteroidetes, and Proteobacteria were the second (25.0%) and the third (22.5%) abundant phylum, respectively. Firmicutes usually is the dominant phylum in cellulose degradation system (Kröber et al., 2009; Eichorst et al., 2013). Therefore, that Firmicutes dominated sample XM3 might be ascribed to the feeding substrate of kitchen waste which is rich in cellulose.

Within Proteobacteria, Alpha-, Beta-, and Gamma-proteobacteria showed similar abundance (averaging 17.7, 17.1, and 16.0%, respectively) in the 7 EAB samples. Though, showing similar average abundance in all samples, Alpha- and Gamma-proteobacteria had extremely high abundance (>60%) in XM1 and BJ-CZ, respectively. Deltaproteobacteria was usually reported as the most dominant class in anodic EAB. However, the class occurred at relative low levels (0.1–10.7, averaging 4.5%) in this study, which was considerably different from previous studies with very low pyrosequencing depth (Lee et al., 2010; Yates et al., 2012).

It is interesting that Epsilonproteobacteria showed very low abundance in 6 samples (0–0.5%) except for CS-LXM (8.2%) which was fed with excess sludge. Previous studies showed that Epsilonproteobacteria occurred at very low level (<1%) in activated sludge (Zhang et al., 2012), anodic biofilm (Lee et al., 2010; Miceli et al., 2012; Yates et al., 2012), and excess sludge anaerobic digester (Zhang et al., 2009). The result in this study seemed to show the ability of BES to enrich some strains in Epsilonproteobacteria for excess sludge degradation.

Besides Alpha-, Beta-, Delta-, and Gamma-proteobacteria, two classes of Bacteroidia and Clostridia were dominant and commonly shared class (relative abundance >1.0%, occurring in at least 5 of 7 samples; Figure S3). Clostridia, typical cellulose degradators in mesophilic environment, was the most dominant (39.3%) class in sample XM2, which could be attributable to the cellulose rich substrate used in this BES.

### Similarity analysis of the EAB samples

**Figure 2:**
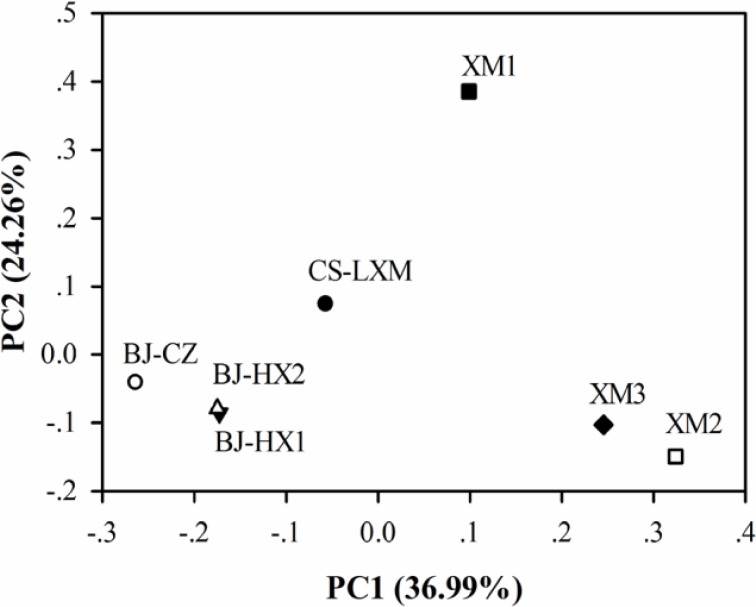
**OTU abundance based principal coordinate analysis of 7 EAB samples using weighted UniFrac**.

Using OTUs abundance, principal coordinate analysis (PCoA) using weighted UniFrac approach was conducted to compare the similarities between the 7 EAB samples (Figure 2). Though being fed with sodium acetate, the three samples from Beijing were different from XM1 and XM3. Some research indicates significant differences between the microbial communities in sewage treatment plants from different geographical areas (Zhang et al., 2012). Since most of the EAB was inoculated with activated sludge, the PCoA analysis demonstrates that the geographical areas other than the substrates significantly influenced the microbial communities in EAB. The close relationship of samples BJ-HX1 and BJ-HX2 also indicated high repeatability and reliability of the pyrosequencing. Results from OTU based cluster analysis (Figure S4) well agreed with the PCoA analysis.

### Core genera

There were 275 OTUs that could be assigned at genera level (Table S2). OTU number was significantly less than that reported in activated sludge (Zhang et al., 2012), indicating that there are many unknown bacteria in EAB. Among these genera, only 20 genera (accounting for 3.6–76.0% of total sequences) could be found in all the 7 samples, and a total of 81 genera (accounting for 7.9–83.4% of total sequences) could be classified as commonly shared genera which were shared by at least 5 samples (Table S3).

**Figure 3:**
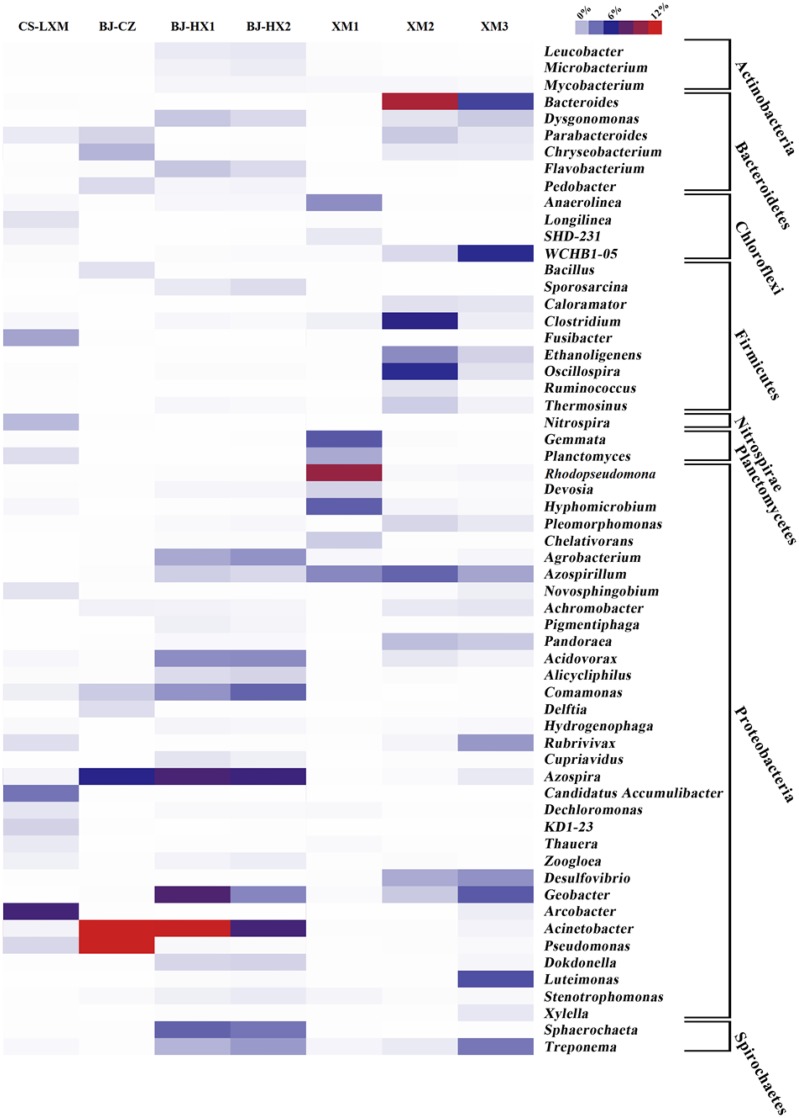
**Heat map of 60 genera whose average relative abundance in 7 EAB samples was higher than 0.1% (a total of >0.71%)**. The color intensity in each panel shows the relative abundance (in percentage) of a genus in a sample, referring to color key at the right top.

There were 60 abundant genera of 275 assigned genera, whose average relative abundance in 7 EAB samples was higher than 0.1% (a total of >0.71%; Figure 3), and 33 genera belong to Proteobacteria. Though, there were 81 commonly shared genera, only 47 genera showed high abundance (a total of >0.71%) in these samples. Among the 60 abundant genera, genera *Azospirillum* (0–4.3%, averaging 1.8%) and *Geobacter* (0–7.5%, averaging 2.4%) showed higher abundance (>1%) in five and four samples, respectively.

Genus *Azospirillum* is reported to be able to fix molecular nitrogen under microaerophilic conditions (Tarrand et al., 1978) and have been rarely detected in bioelectrochemical systems (Hou et al., 2011; Pisciotta et al., 2012). However, a recent study reports (42) that a novel species of *Azospirillum humicireducens* can reduce extracellular anthraquione-2,6-disulfonate, which is an analog of humic substances and known to function as electron shuttle for the bioreduction of U(VI) (Jeon et al., 2004). Though no study has directly reported *Azospirillum* as EAMs, the results seemed to predict their important roles in anodic communities by using electron shuttles e.g., humic substances to perform extracellular electron transfer.

As a model of extracellular electron transfer, genus *Geobacter* is one of the most widely reported EAMs (Lovley, 2012). In some previous studies, *Geobacter* was detected as the most dominant microorganisms in anodic community by low depth pyrosequencing (Lee et al., 2010; Miceli et al., 2012; Yates et al., 2012). With a much deeper sequencing depth in this study, genus *Geobacter* was detected in 6 but not all anodic samples, and only in 4 samples its abundance was >1%. The results indicated that *Geobacter* was not always the dominant microorganisms in anodic communities of BESs, and low sequencing depth would have introduced inaccurate conclusions in analyzing complex microbial communities.

There were four genera i.e., *Acinetobacter, Bacteroides, Pseudomonas*, and *Rhodopseudomonas* which dominate one or two samples with very high abundance (>10%). (i) Genus *Acinetobacter*, reported as nosocomial pathogens (Bergogne-Bérézin and Towner, 1996), was detected as dominant bacteria in samples BJ-CZ and BJ-HX1 (abundance >7% in BJ-HX2). Based on clone libraries, previous studies have detected the genus as abundant bacteria in anodic (Sun et al., 2010) or cathodic communities (Rabaey et al., 2008). Together with all these results, *Acinetobacter* was suggested to be an important composition in EAB community and further electrochemical test might be helpful to understand their roles in EAB. (ii) A previous study has once isolated a Fe(III)-reducing fermentative bacterium *Bacteroides* sp. W7 from the anode suspension of a BES (Wang et al., 2010). A Fe(III)-reducing usually suggests the potential of extracellular electron transfer. This genus was the most dominant genus in sample XM2 which was fed with kitchen waste and the abundance of this genus decreased as sodium acetate was used as substrate again. Therefore, it's speculated that *Bacteroides* might be very important for BESs' current generation with complex substrate. (iii) It's very interesting that genus *Pseudomonas* accounts for 56.4% of total sequences in sample BJ-CZ. Though some studies have reported *Pseudomonas aeruginosa* as an EAM by secreting electron shuttle of pyocyanin (Rabaey et al., 2005), no research have ever reported such a dominance of *Pseudomonas* in BESs. The results might also suggest mediated extracellular electron transfer mainly contributing to current generation in BJ-CZ. (iv) Genus *Rhodopseudomonas* consists of a group of photosynthetic bacteria, and a strain of *Rhodopseudomonas palustris* can directly produce current coupling with acetate respiration (Xing et al., 2008).

Besides the six genera mentioned above, genus of *Azospira* (formally *Dechlorosoma*) which was detected in all the 7 samples seemed to be another core genus in anodic EAB. Though, the genus was not the most abundant genus in any sample, it showed high abundance (>5%) in BJ-CZ, BJ-HX1, and BJ-HX2. The genus was previously detected as dominant residents in anodic EAB (Sun et al., 2011). Therefore, further investigation on the electrochemical activity of *Azospira* spp. is an urgent need for understanding their roles in EAB due to the absent research on this aspect.

The genus *Shewanella* contains several species of EAMs (especially *Shewanella oneidensis*) and therefore has been widely investigated (Borole et al., 2011). However, the pyrosequencing detected no sequence related to this genus, which suggested that *Shewanella* was not a widely spread EAM. The results from the pyrosequencing showed that some commonly shared genera i.e., *Azospira, Azospirillum, Acinetobacter, Bacteroides, Geobacter, Pseudomonas*, and *Rhodopseudomonas* dominate the bacteria community and therefore were defined as core genera in EAB. Though, the electron transfer of *Geobacter* has been widely studied, very limited attention has been paid to the other genera. The results suggest that, to better understand the electrochemical activity of EAB, the research community should conduct some extra studies to investigate the electron transfer of the bacteria in the other 6 genera.

### Core OTUs

A total of 31502 OTUs (3% distance) were detected in the present study. Among these genera, only 9 OTUs were found in all the 7 samples, and a total of 115 OTUs (accounting for 1.3–38.4% of total sequences) could be classified as commonly shared OTUs which were shared by at least 5 samples (Table S4).

The pyrosequencing detected 108 OTUs with an average relative abundance higher than 0.1%, which accounted for 18.1–81.7% of total sequences (Table S5). While 17, 21, 6, and 15 of 108 OTUs belong to classes Alpha-, Beta-, Delta-, and Gamma-proteobacteria, respectively, only 1 OTU belongs to Epsilonproteobacteria. Besides, 12 and 13 of 108 OTUs belong to classes Bacteroidia and Clostridia, respectively.

**Figure 4:**
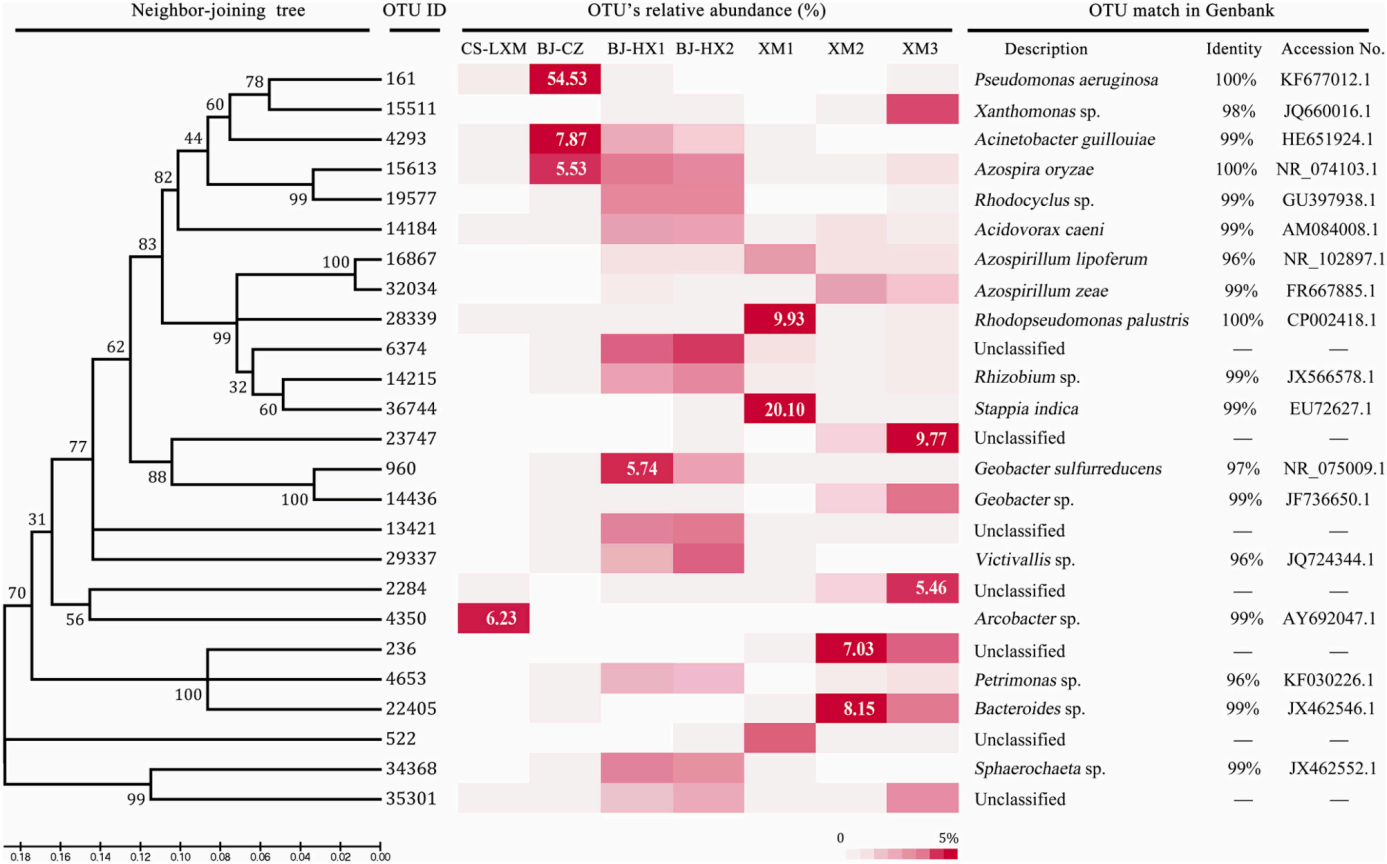
**Relative abundance of the core OTUs (3% distance) in the sequenced 16S rRNA gene sequences**.

Though six genera have been proposed as core genera in EAB, specific species was still unknown. Therefore, a total of 25 OTUs, average relative abundance >0.5%, were selected and designated as core OTUs for further analysis (Figure 4).

Fifteen of 25 OTUs belong to phylum Proteobacteria, and 6 OTUs belong to Alphaproteobacteria. OTU 36744 was the most abundant OTU (abundance of about 20%) in sample XM1. The OTU was 99% identical to strains of *Stappia indica* (GenBank accession number of EU726271.1), which has never been reported as an EAM. OTU 28339 was 99% identical to strain *Rhodopseudomonas palustris* DX-1 which was reported as an EAM (Xing et al., 2008). The OTU was shared by all 7 samples, suggesting that the photosynthetic strain is a widely spread EAM in anodic EAB. Though, OTU 6374 showed high abundance (abundance of 4–5%) in samples BJ-HX1 and BJ-HX2, it could be classified to any specific genus. OTU 14215 was highly identical to strains of *Rhizobium* sp. (99%) or *Agrobacterium tumefaciens* (98%) which both were detected in anodic biofilm of microbial fuel cell (Ishii et al., 2008; Sun et al., 2010). OTUs 16867 and 32034 were highly identical to *Azospirillum lipoferum* (96%) and *Azospirillum zeae* (99%), respectively. As previously discussed, these strains may be able to use electron shuttles for extracellular electron transfer, which still needs further confirmation.

Three OTUs belong to Betaproteobacteria, and OTUs 14184, 15613, and 19577 were highly identical to *Acidovorax caeni* (99%), *Azospira oryzae* (100%), and *Rhodocyclus* sp. (99%), respectively. Though, all the three known genera/species were detected in anodic EAB (Borole et al., 2009; Song et al., 2012), no extracellular electron transfer in these species has been reported (Xiao et al., 2013b). Two OTUs within Deltaproteobacteria i.e., 960 and 14436 shared high similarity to *Geobacter sulfurreducens* strain PCA (97%) and *Geobacter* sp. (99%), respectively. *Geobacter sulfurreducens* strain PCA is known as the model strain for studying bioelectricity generation (Bond and Lovley, 2003), and genus *Geobacter* is one of the most widely reported EAMs (Lovley, 2012). While OTU 960 was a dominant OTU (2.3–5.7%) in BJ-HX1 and BJ-HX2, OTU 14436 was abundant in samples XM2 (0.9%) and XM3 (3.7%). The results seemed to show a geography discrepancy in *Geobacter* species. OTU 4350 belong to Epsilonproteobacteria and shared 99% similarity to uncultured *Arcobacter* sp. Though, this OTU was the most dominant one (abundance of 6.2%) in sample CS-LXM, it was not detected in the other six samples, which might be attributable to the substrate of excess sludge used in this BES. Three OTUs i.e., 161, 4293, and 15511 belong to Gammaproteobacteria. OTU 161 was 100% identical to *Pseudomonas aeruginosa* which was reported as a producer of electron shuttle of pyocyanin (Rabaey et al., 2005). The OTU accounted for more than 54.5% of total sequences in sample BJ-CZ, which confirmed our previous speculation that mediated extracellular electron transfer mainly contributed to current generation in this BES. OTU 4293, sharing 98% similarity to *Acinetobacter guillouiae*, was the second abundant OTU (abundance of 7.9%) in BJ-CZ and also shared high abundance in BJ-HX1 (abundance of 2.0%) and BJ-HX2 (abundance of 1.1%), which indicated that the OTU might be important to the current generation. A strain Ax-9 of *Acinetobacter guillouiae* could degrade synthetic dye (Ng et al., 2014), indicating that the species of bacterium may be electrochemically active.

OTUs 236, 4653, and 22405 belong to class Bacteroidia. OTUs 4653 and 22405 were 96 and 99% identical to *Petrimonas* sp. and *Bacteroides* sp., but OTU 236 did not share high similarity to any specific genus. Previous studies have classified some strains in *Bacteroides* sp. as EAMs (Xiao et al., 2013b), indicating that OTU 22405 relating strain might be important to the current generation of EAB as it accounted for 3.5 and 8.1% of total sequences in XM3 and XM2, respectively. OTUs 29337 and 34368 were 96 and 99% identical to *Victivallis* sp. and *Sphaerochaeta* sp., respectively. Though, strains close to these genera have been detected in microbial fuel cells, no EAM has been reported yet. The left 5 OTUs did not share >95% similarity to known genus, though their abundance was higher than 2% in one or two samples. The results indicated that a great number of dominant strains in EAB are still not isolated and identified.

### Electrochemical tests

**Figure 5:**
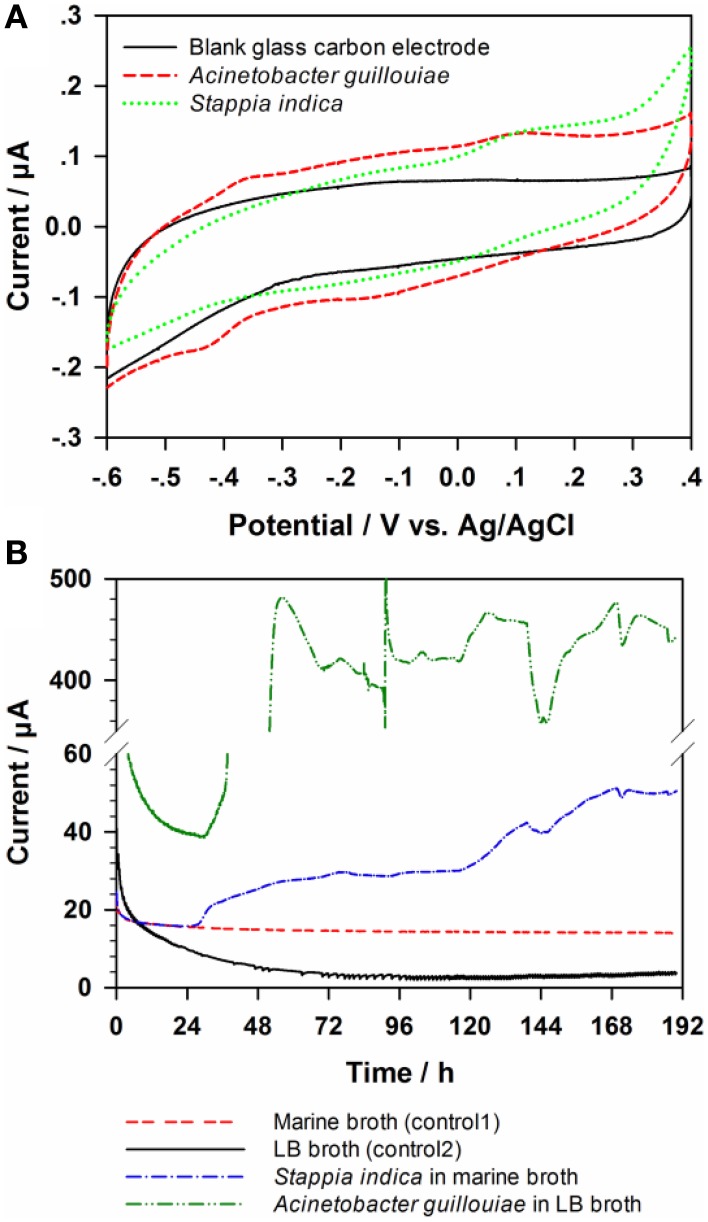
**Cyclic voltammetry (A) and chronoamperometry (B) tests for ***Stappia indica*** and ***Acinetobacter guillouiae*****.

The redox peaks in the CVs of the two bacteria (Figure 5A) provide the first indications that both *Acinetobacter guillouiae* Ax-9 and *Stappia indica* MCCC 1A01226 may be electrochemically active. While four redox peaks (at −0.41, −0.38, −0.14, and +0.95 V vs. Ag/AgCl, respectively) could be counted in the CV of *Acinetobacter guillouiae*, only one oxidation peak at +0.12 V vs. Ag/AgCl could be observed in the CV of *Stappia indica*.

Since redox peaks in a CV don't guarantee bio-electricity production, we employed CA to test the current yield by the bacteria (Figure 5B). The background current of LB and Marne broth were about 4 and 15 μA, respectively. While *Acinetobacter guillouiae* could produce a current higher than 400 μA, a relative low current of about 50 μA was produced by *Stappia indica*. To summarize, CA measurement and CV tests notably indicated that the both bacteria are electrochemically active and can produce electricity, and the results also implied that core OTUs of 36744 and 4293 were electrochemically active and played important roles in current production in anode.

### Community dynamics following substrate change

**Figure 6:**
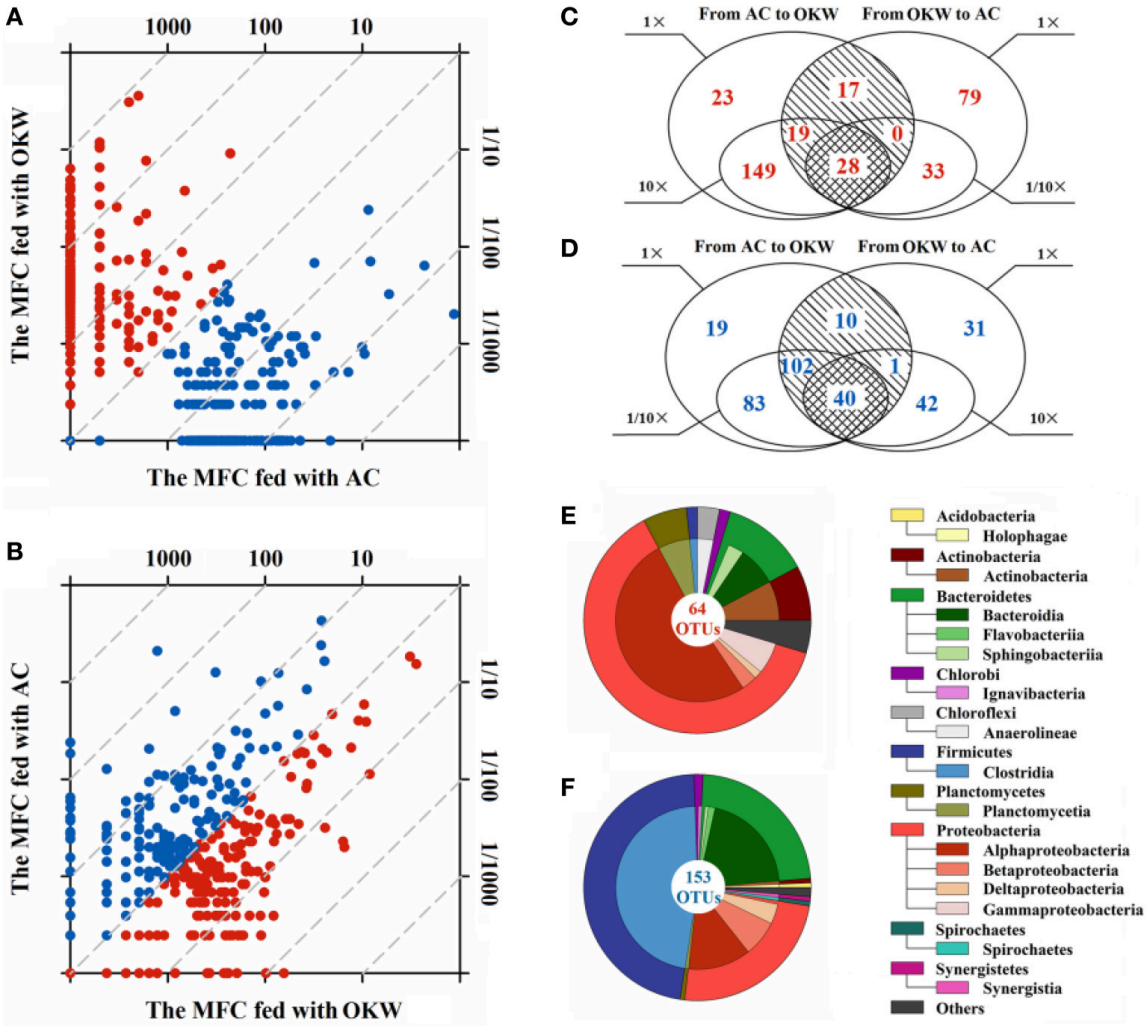
**Scatter plots of 500 dominant OTUs responses to the carbon source of MFCs**. The carbon source of MFC changed from acetate to organic kitchen waste **(A)** and from organic kitchen waste to acetate **(B)**. **(C,D)** Detailed summary of OTUs that showed a significant response to changing carbon source. The 64 OTUs significantly adapted to acetate **(E)** and 153 OTUs significantly adapted to organic kitchen waste **(F)** were analyzed relative to taxonomic assignment. AC, acetate; OKW, organic kitchen waste.

Sample XM1 was collected after the BES was fed with sodium acetate for 8 months. Samples of XM2 and XM3 were collected from the same BES after it was sequentially fed with organic kitchen wastes and sodium acetate for 60 days. The performance of the BES was illustrated in Supporting Material (Figure S5). The relative abundance fold change of OTUs for the substrate of BESs was evaluated as a microbial community dynamics using scatter plots (Figures 6A,B). The scatter plots distinctly showed that 84.20% of OTUs was over 10-fold change or under 1/10-fold change in the first substrate alternation (from acetate to organic kitchen wastes), and 26.80% of OTUs was over 10-fold change or under 1/10-fold change in the second substrate alternation (from organic kitchen wastes back to acetate). The distribution of OTUs was more centralized after the first alternation than that after the second alternation, suggesting microbial community was more impacted by the first substrate alternation. We could infer that the start-up substrate of BESs exerts further influence on substrate changes in the process of operation.

To address the prominent OTUs related to substrate change in the microbial community, we classified those OTUs that upregulated in the first alternation or downregulation in the second alternation as objects which were apt to acetate. Another part of OTUs which adapted to organic kitchen wastes were selected following the similar way which downregulated in the first alternation or upregulation in the second alternation. Above two kinds of OTUs (>1-fold, ≥10-fold change) were figured using Venn diagram between carbon source conditions (Figures 6C,D). 64 OTUs significantly adapted to acetate and 153 OTUs significantly adapted to organic kitchen wastes, suggesting microbial community better adapted to multi-component organic kitchen wastes. Among 64 OTUs, 20 OTUs were not detected by pyrosequencing after the first alteration and were came back after retrieving acetate, which suggesting that OTUs had a certain degree of preference on the substrate.

Based on taxonomic assignment, 7 and 9 dominant phyla were detected in the 64 OTUs and 153 OTUs (Figures 6E,F), respectively. Three dominant phyla (relative abundances over 1%), that was Bacteroidetes (22.22%), Firmicutes (47.06%), Proteobacteria (23.53%), were detected in apt-organic kitchen wastes OTUs. Among apt-acetate OTUs, a wider range of phyla was identified as dominant, i.e., Actinobacteria (7.81%), Bacteroidetes (10.94%), Chloroflexi (3.13%), Firmicutes (1.56%), Planctomycetes (6.25%), and Proteobacteria (62.50%). The dominant portion was changed from Proteobacteria to Firmicutes between apt-acetate OTUs and apt-kitchen garbage OTUs, suggesting the substrate of BESs could drive the change of microbial community. Such as those described genera, *Bacteroides* was abound in XM2 which fed with multicomponent substrate, and *Rhodopseudomona* was a dominant genus in XM1.

### Compare with previous studies on EAB

Most previous studies on microbial community in anodic EAB have been heavily relied on 16S rRNA gene fragments based DGGE and clone library analysis (Jung and Regan, 2007; Sun et al., 2011; Beecroft et al., 2012; Liang et al., 2013). Though, some researchers have applied 454 pyrosequencing to investigate the anodic communities in microbial fuel cells (Lee et al., 2010; Yates et al., 2012), their results are sometimes insufficient to fully profile the microbial communities due to limited sequences obtained in those studies. It should be noted that most pyrosequencing studies on EAB try to understand the complex microbial community under control conditions (i.e., primary inoculum, and substrates etc.; Lee et al., 2010; Yates et al., 2012; Wang et al., 2013). There is no doubt that the experiment using controlled conditions is conducive to explore the function under this specific condition. However, a control experiment is limited for studying EAB in specific BESs. It is necessary from an overall point, to develop the heterogenous BESs for fully understanding EAB.

The study is the first systematic work on the microbial communities of multiple anodic EAB samples by investigating >30,269 and 16S rRNA gene fragments per sample. By employing PCR-based 454 pyrosequencing technique, the deep sequencing showed a high diverse microbial community in anodic EAB and proposed some core genera and OTUs in the 7 geographically separated EAB samples from China.

Some of our findings agree with part of previous research. For example, Proteobacteria were the dominant members in most anodic EAB samples, and Epsilonproteobacteria were the rarely existed bacteria (Lee et al., 2010; Yates et al., 2012). However, our study showed below novel findings: (1) extremely diverse bacterial communities were found in anodic EAB samples, and Deltaproteobacteria was not the dominant class in all samples, the results should be attributable to the deeper sequencing depth used in this study. (2) Several genera of *Azospira, Azospirillum, Acinetobacter, Bacteroides, Geobacter, Pseudomonas*, and *Rhodopseudomonas* played roles as core members in randomly selected anodic EAB samples, and studies on their EET, rather than confined to *Geobacter*, will help us understand the electron transfer within mixed bacterial EAB. The genus of *Shewanella*, one of the model strains for electron transfer studies, was not detected in all EAB samples. (3) The study showed that some pathogens i.e., *Acinetobacter* spp. and *Pseudomonas aeruginosa* were highly dominant members in some samples, which reminds the research community to be very careful in conducting research.

## Conclusions

Using randomly selected EAB samples and pyrosequencing technology, we have profiled a diverse bacterial community in anodic biofilm of BES. Six commonly shared and abundant genera i.e., *Azospira, Azospirillum, Acinetobacter, Bacteroides, Geobacter, Pseudomonas*, and *Rhodopseudomonas* were defined as core genera in electrochemically active communities. Twenty-five OTUs were selected as core OTUs in EABs, and two core OTUs related strains from *Stappia indica* and *Acinetobacter guillouiae* were proven to be electrochemically active. Most dominant bacteria in EAB may be electrochemically active, and high-throughput sequencing such as pyrosequencing is helpful to suggest potential EAMs.

## Author contributions

Idea: YX; data analysis: YX, YZ, SW, and FZ; data collection: YX, YZ, SW, EZ, ZC, PL, XH, and FZ; writing and reviewing the manuscript: all authors.

### Conflict of interest statement

The authors declare that the research was conducted in the absence of any commercial or financial relationships that could be construed as a potential conflict of interest.
