# Supplementary material for: Pyrosequencing Reveals a Core Community of Anodic Bacterial Biofilms in Bioelectrochemical Systems from China
Source: Front Microbiol. 2015 Dec 16;6:1410. doi: 10.3389/fmicb.2015.01410 (PMC4679932; doi:10.3389/fmicb.2015.01410)
Supplement: Supplementary file 1 [file Presentation1.PDF]

Supporting Information

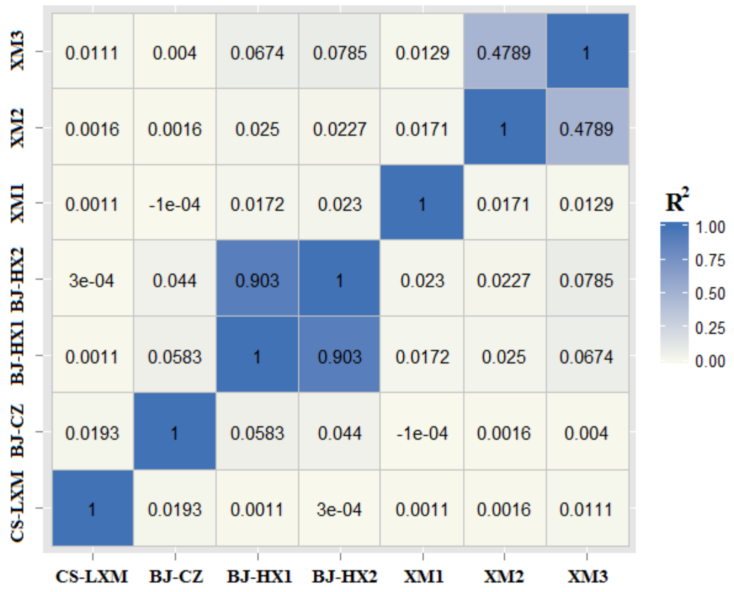

Figure S1 Pearson correlation coefficient for the 7 anodic EAB samples.

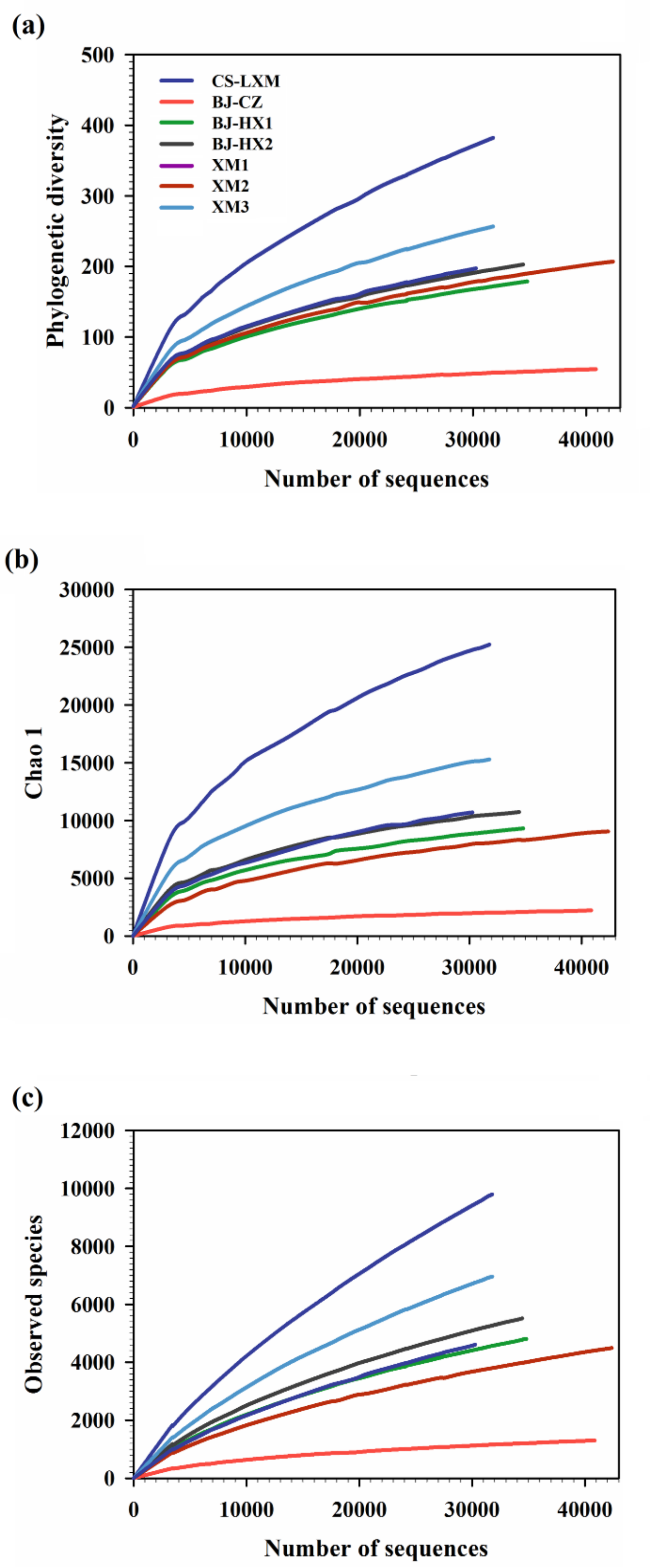

Figure S2 Plots of Phylogenetic Diversity Index (a), Chao 1 (b) and OTU number (c) versus sequence numbers.

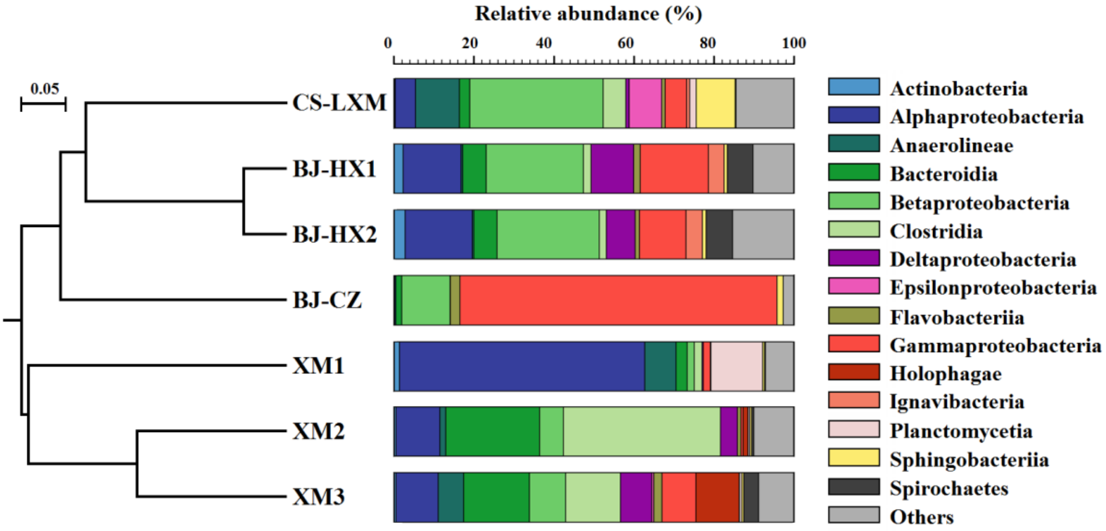

Figure S3 Relative abundances of major classes (>1% in at least one sample) in the 7 anodic EAB samples.

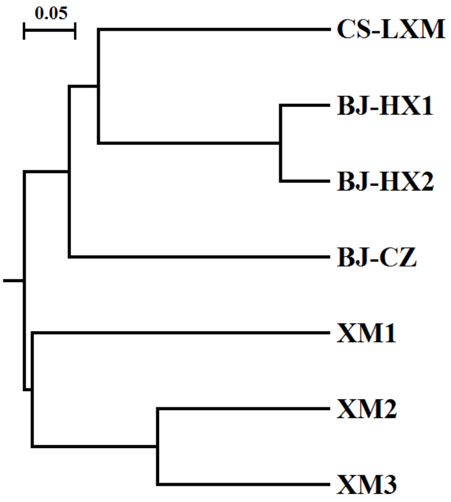

Figure S4 OTU abundance based cluster analysis of 7 EAB samples.

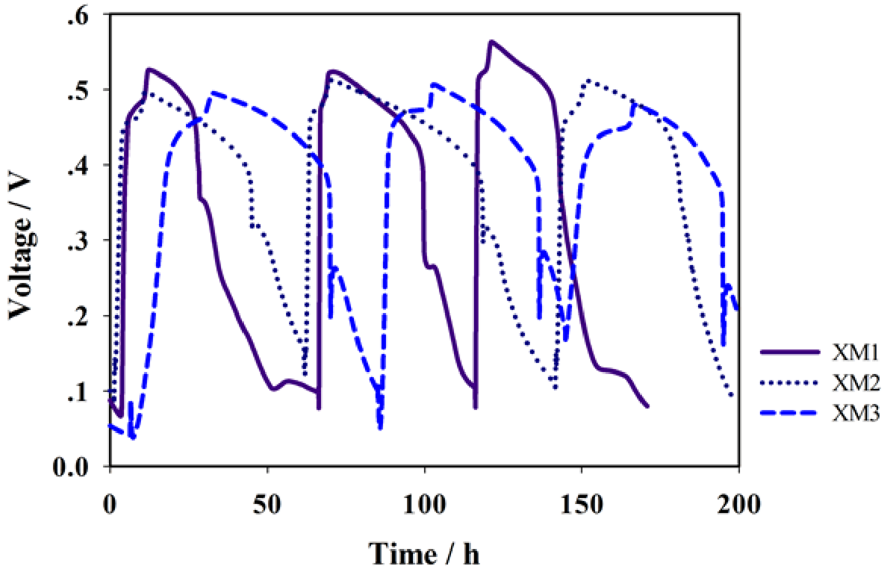

Figure S5 Voltage of the BES before collecting samples XM1, XM2 and XM3.

Table S1 Raw, denoised and effective reads, plus numbers of OTUs, Chao 1, PD index, and GOOD's coverage of 7 EAB samples

**EAB sample**		**Read**			**3% cutoff (per 30 269 sequences)**			
Code	Source (name, city and location)	Raw	Denoised	Effective	OTUs	Chao1	PD index	Good's coverage (%)
CS-LXM	Prof. Xiao-Ming Li, Changsha, south-central China	35474	2330	33144	9468	24778	372.36	78.4
BJ-CZ	Dr. Zheng Chen, north China	43539	1331	42208	1129	1982	48.27	98.2
BJ-HX1	Prof. Xia Huang, north China	37646	2584	35062	4434	8859	168.30	91.5
BJ-HX2		37446	3004	34442	5119	10357	191.46	89.9
XM1	Dr. Yong Xiao, Xiamen, southeast China	47360	3492	43868	3695	7972	178.71	92.7
XM2		33901	1669	32232	6751	15117	250.89	85.7
XM3		31425	1156	30269	4607	10699	197.26	90.2

Table S2 Assigned genera in the 7 EAB samples

Taxonomy		Relative abundance						
**Phylum**	**Genus**	**CS-LXM**	**BJ-CZ**	**BJ-HX1**	**BJ-HX2**	**XM1**	**XM2**	**XM3**
Acidobacteria	Candidatus *Solibacter*	0.000875	4.7384E-05	0.0005134	0.00029	0.000251	0.0005274	0.001784
	Candidatus *Microthrix*	0	0	0	5.81E-05	0	0	0
	Brevibacterium	0	0	0	5.81E-05	0	6.205E-05	6.61E-05
	Actinotalea	0	0	8.556E-05	5.81E-05	0	0	0
	Cellulomonas	0	2.3692E-05	0	0	0	9.308E-05	0.000132
	Corynebacterium	0	0	0.0001141	0	0	0	0
	Brachybacterium	0	0	0	0	0	3.103E-05	0
	Dietzia	0	0	0.0001711	0.000203	0.001778	9.308E-05	9.91E-05
	Gordonia	0	2.3692E-05	0.0015401	0.002439	0.001049	0.0001241	0.000264
	Phycicoccus	6.034E-05	0	0	0	0	0	0
	Tetrasphaera	0.000181	2.3692E-05	0	0	0	0	0
	Agromyces	0	9.4769E-05	0	0	0	0	0
	Leucobacter	6.034E-05	4.7384E-05	0.0054475	0.006155	0.000479	0.0001241	0
	Microbacterium	9.051E-05	0.00023692	0.0033655	0.004849	0.00057	0.0001241	0.000231
Actinobacteria	*Yonghaparkia*	0	0	0	0	0.003625	0	0
	Arthrobacter	0	0	8.556E-05	0	0	0	0
	Nesterenkonia	0	0	0	0	2.28E-05	0	0
	Mycobacterium	0	0	0.0023387	0.002265	0.001869	0.0017995	0.001222
	Nocardia	0	0	0	0	2.28E-05	0	0
	Rhodococcus	0	2.3692E-05	0.0019394	0.002061	0.00057	6.205E-05	0.000165
	Aeromicrobium	0	0	2.852E-05	5.81E-05	0	0	0
	Propionicimonas	6.034E-05	0	0.0007701	0.001684	0.000388	0.0009928	0.00152
	Cellulosimicrobium	0	0	0	0	6.84E-05	3.103E-05	3.3E-05
	Microlunatus	0	2.3692E-05	0	0	0	0	0
	Tessaracoccus	3.017E-05	0	0	0	0	0	0
	Amycolatopsis	0	0	2.852E-05	0	0	0	0
	Pseudonocardia	0	0	0	0	0	3.103E-05	9.91E-05
	Tsukamurella	0	0	0.0009412	0.000813	0	0	0
Armatimonadetes	*Fimbriimonas*	6.034E-05	0	0	0	0	0	0
	Chthonomonas	3.017E-05	0	0	0	0	0	0
	Odoribacter	0	0	0	0	2.28E-05	0	0
	Bacteroides	0.0004526	4.7384E-05	0	0	0.000114	0.108929	0.051439
	Dysgonomonas	0	7.1077E-05	0.014945	0.010133	0.000182	0.0077252	0.014206

	Parabacteroides	0.0054007	0.0115381	0	2.9E-05	0	0.0146438	0.006508
	Chryseobacterium	0.0001207	0.02018575	8.556E-05	0.000319	0	0.0056466	0.005253
	Elizabethkingia	3.017E-05	0	0	0	0	0	0
	Flavobacterium	0.0004827	0.00056861	0.0153728	0.009726	6.84E-05	3.103E-05	0
	Haloanella	0	0	2.852E-05	0	0	0	0
	Kaistella	3.017E-05	0	0.0004849	0.000232	6.84E-05	0.0005895	0.000132
	Myroides	0	0	5.704E-05	5.81E-05	0	0	0
	Riemerella	0.0013577	0	0	0	0	0	3.3E-05
Bacteroidetes	*Wautersiella*	0	0	2.852E-05	0	0	0	0
	Chitinophaga	3.017E-05	0	5.704E-05	2.9E-05	0	3.103E-05	3.3E-05
	Niabella	0	0	8.556E-05	5.81E-05	0	0.0005895	9.91E-05
	A4	0.0004526	0	0	0	0.000684	0	3.3E-05
	Dyadobacter	0	0	8.556E-05	0.000203	0.00041	0	0.000165
	Emticicia	0	0	0	0	0.000502	0	0
	Runella	0.0028361	0	0	0	2.28E-05	0	0
	Haliscomenobacter	0.0004827	0	0	0	0	0	0
	Parapedobacter	0	0	2.852E-05	0	0	0	0
	Pedobacter	6.034E-05	0.00973749	0.0023672	0.002991	0	0	0
	Sphingobacterium	3.017E-05	0.00495167	0	2.9E-05	0	0.0001241	0.000198
Chlorobi	*Ignavibacterium*	9.051E-05	0	0	0	0.001185	0.0001241	9.91E-05
	Anaerolinea	0.0017801	2.3692E-05	0.0019394	0.001974	0.030957	0.0002172	9.91E-05
	Bellilinea	0	0	0	0	0.001664	0	0
	C1_B004	0	0	0	0	0.00155	0	0
	Longilinea	0.0079652	0	0	0	0.000296	0	0
Chloroflexi	*SHD-14*	0.0028663	0	0	2.9E-05	0.000342	3.103E-05	0
	SHD-231	0.0034094	0	0	2.9E-05	0.005927	0	0
	T78	0.0043447	0	2.852E-05	0	0.000684	0	0
	WCHB1-05	0.0005733	0	0.0006275	0.000871	0.000957	0.0101452	0.058178
	Caldilinea	0.0053403	0	0	0	0	0	0
	Kouleothrix	0.000875	0	0	0	0	0	0
Fibrobacteres	*Fibrobacter*	0	0	0.0010553	0.000813	2.28E-05	0	0
	Bacillus	9.051E-05	0.00779473	2.852E-05	8.71E-05	0.000137	0	0
	Aneurinibacillus	0	0	0	0	0	0	3.3E-05
	Cohnella	0	2.3692E-05	0	2.9E-05	0	0	0
	Kurthia	6.034E-05	0	0	0	0	0	0
	Lysinibacillus	0.0002715	0	0	0	0	0	0
	Sporosarcina	0	4.7384E-05	0.0055616	0.009233	2.28E-05	0	0

	Exiguobacterium	3.017E-05	0	0	0	0	0	0
	Lactobacillus	9.051E-05	0	0	0	0	0	0
	Leuconostoc	3.017E-05	0	0	0	0	0	0
	Turicibacter	0	0	0	0	0	0	3.3E-05
	Acidaminobacter	0.0005733	0	0	0	4.56E-05	0	0
	Caloramator	0.000181	0	0	0	0.00016	0.0080665	0.006806
	Clostridium	0.0016594	0	0.0017968	0.001278	0.003944	0.0626396	0.004691
	Fusibacter	0.0250121	4.7384E-05	0	0	0.00016	0	0
	Natronincola	0	0	0	0	2.28E-05	0	0
	Sedimentibacter	9.051E-05	0	0.0005134	0.000523	2.28E-05	0.0004033	9.91E-05
	Dehalobacterium	0	0	0	0	0	9.308E-05	6.61E-05
	Roseburia	3.017E-05	0	0	0	0	0	0
Firmicutes	*Shuttleworthia*	3.017E-05	0	0	0	0	0	0
	Desulfitobacterium	0	0	0	2.9E-05	0	0	0
	Desulfosporosinus	0	0	0	0	0	3.103E-05	6.61E-05
	Niigata-25	3.017E-05	0	0	0	0	0	0
	Pelotomaculum	0	0	0	0	0	0	6.61E-05
	Anaerofilum	0	0	0	0	0	0.0001551	0.000132
	Ethanoligenens	0	0	0.0001141	0.000174	0.000137	0.0315215	0.012092
	Oscillospira	0.0003319	0	0.0006275	0.000581	0.000182	0.0578307	0.007896
	Ruminococcus	0.0001509	0	0	0	0	0.0076322	0.000727
	Syntrophomonas	0	0	0	2.9E-05	0	0	0.001123
	Acidaminococcus	0	0	2.852E-05	0	0	0	0
	Desulfosporomusa	0	0	0	0	0	0.0017374	9.91E-05
	Mitsuokella	6.034E-05	0	0	0	0	0	0
	Phascolarctobacterium	0.0002414	0	0	0	0	0	0
	Propionispora	0	0	0	0	0	0.0029164	0.000297
	Thermosinus	3.017E-05	0	0.0017683	0.001132	2.28E-05	0.0132787	0.003469
	vadinHB04	0.000181	0	0	5.81E-05	0	0	0
	KF-Gitt2-16	0	0	0	0	2.28E-05	6.205E-05	0
	Bulleidia	3.017E-05	0	0	0	0	0	0
	PSB-M-3	0.0006939	0	0	0	0.001003	0	0
Gemmatimonadetes	*Gemmatimonas*	0	0	0	0	4.56E-05	0.0004344	0.00152
Nitrospirae	*Nitrospira*	0.0187968	4.7384E-05	0	0	0	0	0
	Gemmata	0.0004526	0	0	2.9E-05	0.045888	0.0007756	9.91E-05
	Isosphaera	0	0	0	0	0	0	3.3E-05
Planctomycetes	*Singulisphaera*	0	0	0	0	2.28E-05	3.103E-05	0

Planctomycetes	*A17*	6.034E-05	0	0	0	0.005425	3.103E-05	3.3E-05
	Pirellula	9.051E-05	0	0	0	0	0	0
	Planctomyces	0.0090514	0	0	0	0.023366	0.0001551	3.3E-05
	Arthrospira	0	0	2.852E-05	5.81E-05	2.28E-05	0	0
	Brevundimonas	0	2.3692E-05	0.0002282	0.000203	0.000342	0	0.000529
	Caulobacter	0	0	0	0	0	6.205E-05	0.000264
	Phenylobacterium	0.0001207	0	0.0001141	2.9E-05	0.001869	0.0013031	0.001255
	Bartonella	0	2.3692E-05	0	8.71E-05	0	0	0
	Chelatococcus	3.017E-05	0	0	0	0	0	0
	Afipia	0	0	0	0	0.000182	3.103E-05	0
	Bosea	6.034E-05	2.3692E-05	0.0003423	0.000552	0.101714	0.0016133	0.002379
	Bradyrhizobium	3.017E-05	0	0	0	0.000274	0	0.000363
	Ochrobactrum	0	0.00037908	0.0004278	0.000348	0.001664	6.205E-05	3.3E-05
	Devosia	0.0005431	4.7384E-05	0.0022246	0.002178	0.011877	0.0005274	0.000925
	Hyphomicrobium	0.0019611	0	0.0001996	0.000319	0.04411	0.0029474	0.001255
	Parvibaculum	0	0	0.0001711	0.000581	0.00098	0.0013341	0.001553
	Pedomicrobium	0.0001509	0	0	0	9.12E-05	0	0
	Rhodoplanes	3.017E-05	0	0	0	0.00098	0.0001241	0.000165
	Methylosinus	9.051E-05	0	0.0001711	0.000116	0.003738	0.0002792	0.000132
	Pleomorphomonas	6.034E-05	2.3692E-05	0.0011979	0.0018	4.56E-05	0.0110449	0.006013
	Aminobacter	0	0	0	0	9.12E-05	3.103E-05	3.3E-05
	Chelativorans	0	2.3692E-05	0.000656	0.000639	0.013609	0.0001241	0.000132
	Defluvibacter	3.017E-05	0.00018954	0.0004849	0.000494	0.000114	0	3.3E-05
	Mesorhizobium	0	0	0	0	9.12E-05	0	0
	Phyllobacterium	0	0	0	2.9E-05	0	0	0
	Pseudaminobacter	0	0	0	0	0	3.103E-05	0
	Agrobacterium	0	7.1077E-05	0.0236153	0.029731	0.002006	0.0004654	0.002346
	Kaistia	0	0	2.852E-05	0	0.000228	0.0006515	0.001949
	Rhizobium	0	0	0	2.9E-05	0	0	3.3E-05
	Shinella	3.017E-05	2.3692E-05	0.0001711	5.81E-05	0.000114	3.103E-05	3.3E-05
	Azorhizobium	0	0	0	0	0	0	6.61E-05
	Xanthobacter	0	0	0.0002852	0.000232	9.12E-05	0.0002172	0.000496
	Hyphomonas	0	0	0	0	0.003921	6.205E-05	0
	Oceanicaulis	0	0	0	0	2.28E-05	0	0
	Paracoccus	9.051E-05	0	5.704E-05	0.000116	0.000547	9.308E-05	0.000198
	Rhodobacter	0.0017198	0	0	0	0.001117	0	0
	Roseomonas	0.0007845	0	8.556E-05	2.9E-05	0.000365	0.0013651	0.001288

	Tanticharoenia	3.017E-05	0	0	0	0	0	0
	Azospirillum	0	0.00047384	0.0131481	0.010307	0.032643	0.0425354	0.024646
	Inquilinus	0	0	0	0	6.84E-05	3.103E-05	0
	Magnetospirillum	0	0	5.704E-05	0.000174	9.12E-05	0.0008997	0.00076
	Novispirillum	0.0003017	0	0	0	0	0	0
	Oleomonas	0	0	0	0	0.000707	0.0006515	0.003072
	Phaeospirillum	0	0	0.0001711	0.000203	4.56E-05	0.0006515	0.000925
	Telmatospirillum	0	2.3692E-05	0.003451	0.002265	2.28E-05	0	9.91E-05
	Erythromicrobium	0	0	0	0	0	3.103E-05	0
	Novosphingobium	0.0076937	9.4769E-05	2.852E-05	2.9E-05	2.28E-05	0.0008687	0.004262
	Sphingobium	3.017E-05	0.00078184	0.0002282	0.000406	0.001846	0.0003103	0.000363
	Sphingomonas	0.0001207	2.3692E-05	2.852E-05	5.81E-05	9.12E-05	6.205E-05	0.00033
	Sphingopyxis	3.017E-05	0	8.556E-05	0.000116	0.001231	0.00121	0.00152
	Achromobacter	0	0.00331691	0.0032514	0.002352	0.000137	0.0057086	0.006872
	Oligella	3.017E-05	0	0	0	0	0	0
	Pigmentiphaga	0	0	0.00405	0.002642	2.28E-05	0.0001241	0.00033
	Tetrathiobacter	0	0	0	2.9E-05	0	0	3.3E-05
	Burkholderia	0	0	0	2.9E-05	0	0.0004033	9.91E-05
	Pandoraea	0	2.3692E-05	0.001426	0.001481	0	0.0177774	0.014371
	Acidovorax	0.0020818	0.00037908	0.0311734	0.031647	9.12E-05	0.0063291	0.003304
	Alicycliphilus	0.0007543	0.00040277	0.009526	0.011556	6.84E-05	0.0008067	0.000165
	Aquabacterium	0.0003621	0	0	0	0	0	0
	Comamonas	0.004224	0.01383624	0.0293195	0.042622	0.000182	0	3.3E-05
	Curvibacter	6.034E-05	0	0	0	0	0	0
	Delftia	0	0.00895565	5.704E-05	0.000116	0	3.103E-05	0.000165
	Giesbergeria	3.017E-05	0	0	0	0	0	0
	Hydrogenophaga	0.0012672	0	0.0027095	0.001887	0.000342	0.0009928	0.001388
	Hylemonella	0.0020517	0	0.0008556	0.000755	0	0	0
	Leptothrix	0.0002414	0	0	0	0	0	0
	Limnobacter	0.0001207	0.00026061	0	0	0	0	0
	Limnohabitans	0.0002414	0	0	0	0	0	0
	Methylibium	0.0010862	0	0	0	0	0	0
	Pelomonas	0	0	0	0	2.28E-05	0	0
	Ramlibacter	0.0003922	2.3692E-05	0.0001141	0	0	0	0
	Rhodoferax	0.0001509	0	0	0	0	0	0
	Rubrivivax	0.0087195	0	8.556E-05	2.9E-05	2.28E-05	0.0027612	0.028445
Proteobacteria	*Simplicispira*	0	0	5.704E-05	2.9E-05	0	0	0

Thiomonas	3.017E-05	0	0	0	0	0	3.3E-05
Variovorax	0	0.00014215	2.852E-05	0	0	0	0
Xenophilus	0	2.3692E-05	0.0022532	0.002178	0	0.0002482	3.3E-05
Cupriavidus	0	0	0.0069591	0.003978	0	0.0003103	0.00033
Oxalobacter	6.034E-05	0	0.0005419	0.00029	2.28E-05	0	0
Ralstonia	0	0	0	0	4.56E-05	3.103E-05	0
Thiobacillus	9.051E-05	0	0	0	0.000114	0.0006205	0.00033
Methylobacillus	0	0	5.704E-05	2.9E-05	0	0	0
Microvirgula	9.051E-05	0	0	0	0	0	0
Vitreoscilla	3.017E-05	0	0	0	0	0	0
Vogesella	3.017E-05	0	0	0	0	0	0
Azospira	0.002987	0.06145754	0.0734128	0.068376	6.84E-05	0.0008687	0.005583
Azovibrio	0	0	2.852E-05	0	0	0	0
Candidatus *Accumulibacter*	0.0381668	4.7384E-05	2.852E-05	0	0	0	0
Dechloromonas	0.0067584	0	0.001312	0.001161	0.001641	0.0002172	0.000165
Dok59	0.0001207	0	0	0	0.00041	9.308E-05	6.61E-05
K82	0	2.3692E-05	0.0009412	0.001103	2.28E-05	0	3.3E-05
KD1-23	0.0120685	2.3692E-05	0	8.71E-05	0	0	0
Methyloversatilis	0.0001207	0	0	0	0	9.308E-05	0
Propionivibrio	0.0006034	0	0	0	0	0	0
Rhodocyclus	9.051E-05	0	2.852E-05	2.9E-05	0.001505	0.0006826	9.91E-05
Sterolibacterium	0	0	0	0	0	3.103E-05	0
Sulfuritalea	0.0035602	0	0	0	0	0	0
Thauera	0.0055817	0	2.852E-05	2.9E-05	0.001299	6.205E-05	0.000132
Uliginosibacterium	0.0002414	0	0	0	0	0	0
Zoogloea	0.0036507	4.7384E-05	0.0030232	0.004587	2.28E-05	0.0008067	0.000264
Bdellovibrio	0.000181	0	2.852E-05	0.000116	0	0	3.3E-05
Desulfobulbus	0.0007543	0	0	0	6.84E-05	0	0
Desulfomicrobium	3.017E-05	0	0	0	0	0	0
Desulfovibrio	9.051E-05	2.3692E-05	0.0002282	0.000261	0.000342	0.0229896	0.029899
Geobacter	0	0.00037908	0.0752667	0.033302	0.000866	0.0144267	0.045426
Anaeromyxobacter	6.034E-05	0	0	0	0	0	0
Nannocystis	9.051E-05	0	0	0	0	0	0
Chondromyces	0	0	2.852E-05	2.9E-05	0	0	0
Desulfococcus	3.017E-05	0	0	0	0	0	0
Desulfomonile	0	0	0	0	2.28E-05	9.308E-05	6.61E-05
Syntrophus	0.0002414	0	0	0	2.28E-05	3.103E-05	3.3E-05

	Syntrophobacter	9.051E-05	0	0	0	0.000319	0.0002172	0.000297
	Arcobacter	0.0715062	4.7384E-05	0	0	0	0	0.004625
	Sulfurospirillum	0.0002112	0	0	0	0	3.103E-05	6.61E-05
	Sulfuricurvum	6.034E-05	0	0	0	0	0	0
	Oceanimonas	0.0005129	0	0	0	0	0	0
	Tolumonas	3.017E-05	0	0	0	0	0	0
	Alishewanella	0.0006336	0	0	0	0	0	0
	Halothiobacillus	6.034E-05	0	0	0	0	0	0
	Citrobacter	0	0	0	0	0	0.0001551	6.61E-05
	Enterobacter	9.051E-05	0	0	0	0	0	0
	Escherichia	0.0001207	0	0	0	0	0	0
	Klebsiella	3.017E-05	0	0	0	0	0	0
	Plesiomonas	3.017E-05	0	0	0	0	0	0
	Aquicella	0	0	0	0	0.004286	3.103E-05	0.000198
	Legionella	0	4.7384E-05	2.852E-05	2.9E-05	0.00057	0.0005895	0.000231
	Crenothrix	0.0002414	0	0	0	0	0	0
	Methylocaldum	9.051E-05	0	0	0	0.002758	0.0002172	0.000264
	Methylomicrobium	0	0	0	0	0.000274	0	0
	Methylomonas	0	0	2.852E-05	0	2.28E-05	0	0
	Halomonas	6.034E-05	0	0	0	2.28E-05	3.103E-05	0
	Marinobacterium	0.0012974	0	8.556E-05	0	0	0	0
	Acinetobacter	0.003168	0.13108889	0.11936	0.071482	0.000319	0.0004033	0.00294
	Psychrobacter	0.0006939	0	2.852E-05	0	0	0	0
	Pseudomonas	0.0107108	0.56406368	0.0015972	0.000639	2.28E-05	0.0004964	0.001123
	Thiothrix	0.0009051	0	0	0	0	0	0
	Steroidobacter	0.0001509	0	0	0	0	6.205E-05	6.61E-05
	Dokdonella	0.0001509	2.3692E-05	0.0108379	0.011788	0.000205	0.0001862	0.00218
	Dyella	0	0	0	5.81E-05	0	0	0
	Luteimonas	0	0	0.000713	0.001161	0.000114	0.0002172	0.048135
	Pseudoxanthomonas	0.000181	0.00054492	0	0	6.84E-05	0.0002172	0
	Stenotrophomonas	6.034E-05	0.00137415	0.00405	0.005517	0.002713	0.0006515	0.001652
	Thermomonas	0.0022327	0	0.0003708	0.000581	2.28E-05	0	0.000562
	Xylella	3.017E-05	0	0.0003993	0.000436	2.28E-05	0.0002172	0.006178
	SJA-88	0	0	0	0	0	3.103E-05	0.000463
	Sphaerochaeta	3.017E-05	0.00016585	0.043038	0.038122	4.56E-05	0	3.3E-05
Spirochaetes	*Spirochaeta*	6.034E-05	0	0	0	0	0	0
	Treponema	0.0015387	0.00011846	0.020421	0.027408	0.00253	0.0053363	0.037299

	za29	0	0	0	0	9.12E-05	3.103E-05	0
	W22	6.034E-05	0	0	0	9.12E-05	0	0
	Aminiphilus	0	0	2.852E-05	0	0.001117	0.0008687	0.00033
	Aminobacterium	6.034E-05	0	0	0	0	0	0
	HA73	0	0	0.0002852	0.000232	0.003807	0.0004654	0.000231
	PD-UASB-13	9.051E-05	0	0	0	0	0	0
Synergistetes	*Cloacibacillus*	0	0	0.0002567	0.00029	0	0	0.000165
	Synergistes	0	0	0	0	0	3.103E-05	6.61E-05
	Thermococcus	0	0	0	0	0	0.0004654	0.000231
	vadinCA02	9.051E-05	0	0.001426	0.000987	0.000137	0.0003413	0.000727
	E6	0.000181	0	0	2.9E-05	0.000251	0.0004654	9.91E-05
Tenericutes	*Acholeplasma*	0.0002112	0	0	0	0.000114	0	0
	RFN20	9.051E-05	0	0	0	0	0	0
Thermi	*Deinococcus*	0	0	0.0001141	5.81E-05	0.00155	0.0001862	9.91E-05
Thermotogae	*Kosmotoga*	0	0	0	0	2.28E-05	3.103E-05	0
	Pedosphaera	0	0	0	0	0	3.103E-05	0
	Candidatus *Xiphinematobacte*	0	0	0.0001141	8.71E-05	0.00057	0	0.000297
Verrucomicrobia	*Opitutus*	0.0001509	0	0.0008556	0.001597	0	0	0
	Luteolibacter	3.017E-05	0	0	0	0	0	3.3E-05
	Prosthecobacter	0.0002112	0	0	0	0.000296	0	0

Table S3 Commonly shared genera which were shared by at least 5 samples

Phylum	Genus	Relative abundance (%)						
		CS-LXM	BJ-CZ	BJ-HX1	BJ-HX2	XM1	XM2	XM3
Acidobacteria	Candidatus *Solibacter*	0.087	0.005	0.051	0.029	0.025	0.053	0.178
Actinobacteria	*Dietzia*	0	0	0.017	0.02	0.178	0.009	0.01
	Gordonia	0	0.002	0.154	0.244	0.105	0.012	0.026
	Leucobacter	0.006	0.005	0.545	0.616	0.048	0.012	0
	Microbacterium	0.009	0.024	0.337	0.485	0.057	0.012	0.023
	Mycobacterium	0	0	0.234	0.226	0.187	0.18	0.122
	Rhodococcus	0	0.002	0.194	0.206	0.057	0.006	0.017
	Propionicimonas	0.006	0	0.077	0.168	0.039	0.099	0.152
Bacteroidetes	*Bacteroides*	0.045	0.005	0	0	0.011	10.893	5.144
	Dysgonomonas	0	0.007	1.494	1.013	0.018	0.773	1.421
	Parabacteroides	0.54	1.154	0	0.003	0	1.464	0.651
	Chryseobacterium	0.012	2.019	0.009	0.032	0	0.565	0.525
	Flavobacterium	0.048	0.057	1.537	0.973	0.007	0.003	0
	Kaistella	0.003	0	0.048	0.023	0.007	0.059	0.013
	Chitinophaga	0.003	0	0.006	0.003	0	0.003	0.003
	Sphingobacterium	0.003	0.495	0	0.003	0	0.012	0.02
Chloroflexi	*Anaerolinea*	0.178	0.002	0.194	0.197	3.096	0.022	0.01
	WCHB1-05	0.057	0	0.063	0.087	0.096	1.015	5.818
Firmicutes	*Bacillus*	0.009	0.779	0.003	0.009	0.014	0	0
	Clostridium	0.166	0	0.18	0.128	0.394	6.264	0.469
	Sedimentibacter	0.009	0	0.051	0.052	0.002	0.04	0.01
	Ethanoligenens	0	0	0.011	0.017	0.014	3.152	1.209
	Oscillospira	0.033	0	0.063	0.058	0.018	5.783	0.79
	Thermosinus	0.003	0	0.177	0.113	0.002	1.328	0.347
Planctomycetes	*Gemmata*	0.045	0	0	0.003	4.589	0.078	0.01
	Brevundimonas	0	0.002	0.023	0.02	0.034	0	0.053
	Phenylobacterium	0.012	0	0.011	0.003	0.187	0.13	0.126
	Bosea	0.006	0.002	0.034	0.055	10.171	0.161	0.238
	Ochrobactrum	0	0.038	0.043	0.035	0.166	0.006	0.003
	Devosia	0.054	0.005	0.222	0.218	1.188	0.053	0.093

Proteobacteria	*Hyphomicrobium*	0.196	0	0.02	0.032	4.411	0.295	0.126
	Parvibaculum	0	0	0.017	0.058	0.098	0.133	0.155
	Methylosinus	0.009	0	0.017	0.012	0.374	0.028	0.013
	Pleomorphomonas	0.006	0.002	0.12	0.18	0.005	1.104	0.601
	Chelativorans	0	0.002	0.066	0.064	1.361	0.012	0.013
	Defluvibacter	0.003	0.019	0.048	0.049	0.011	0	0.003
	Agrobacterium	0	0.007	2.362	2.973	0.201	0.047	0.235
	Shinella	0.003	0.002	0.017	0.006	0.011	0.003	0.003
	Xanthobacter	0	0	0.029	0.023	0.009	0.022	0.05
	Paracoccus	0.009	0	0.006	0.012	0.055	0.009	0.02
	Roseomonas	0.078	0	0.009	0.003	0.036	0.137	0.129
	Azospirillum	0	0.047	1.315	1.031	3.264	4.254	2.465
	Magnetospirillum	0	0	0.006	0.017	0.009	0.09	0.076
	Phaeospirillum	0	0	0.017	0.02	0.005	0.065	0.093
	Telmatospirillum	0	0.002	0.345	0.226	0.002	0	0.01
	Novosphingobium	0.769	0.009	0.003	0.003	0.002	0.087	0.426
	Sphingobium	0.003	0.078	0.023	0.041	0.185	0.031	0.036
	Sphingomonas	0.012	0.002	0.003	0.006	0.009	0.006	0.033
	Sphingopyxis	0.003	0	0.009	0.012	0.123	0.121	0.152
	Achromobacter	0	0.332	0.325	0.235	0.014	0.571	0.687
	Pigmentiphaga	0	0	0.405	0.264	0.002	0.012	0.033
	Pandoraea	0	0.002	0.143	0.148	0	1.778	1.437
	Acidovorax	0.208	0.038	3.117	3.165	0.009	0.633	0.33
	Alicycliphilus	0.075	0.04	0.953	1.156	0.007	0.081	0.017
	Comamonas	0.422	1.384	2.932	4.262	0.018	0	0.003
	Delftia	0	0.896	0.006	0.012	0	0.003	0.017
	Hydrogenophaga	0.127	0	0.271	0.189	0.034	0.099	0.139
	Rubrivivax	0.872	0	0.009	0.003	0.002	0.276	2.844
	Xenophilus	0	0.002	0.225	0.218	0	0.025	0.003
	Azospira	0.299	6.146	7.341	6.838	0.007	0.087	0.558
	Dechloromonas	0.676	0	0.131	0.116	0.164	0.022	0.017
	K82	0	0.002	0.094	0.11	0.002	0	0.003
	Rhodocyclus	0.009	0	0.003	0.003	0.15	0.068	0.01
	Thauera	0.558	0	0.003	0.003	0.13	0.006	0.013
	Zoogloea	0.365	0.005	0.302	0.459	0.002	0.081	0.026

	Desulfovibrio	0.009	0.002	0.023	0.026	0.034	2.299	2.99
	Geobacter	0	0.038	7.527	3.33	0.087	1.443	4.543
	Legionella	0	0.005	0.003	0.003	0.057	0.059	0.023
	Acinetobacter	0.317	13.109	11.936	7.148	0.032	0.04	0.294
	Pseudomonas	1.071	56.406	0.16	0.064	0.002	0.05	0.112
	Dokdonella	0.015	0.002	1.084	1.179	0.021	0.019	0.218
	Luteimonas	0	0	0.071	0.116	0.011	0.022	4.814
	Stenotrophomonas	0.006	0.137	0.405	0.552	0.271	0.065	0.165
	Thermomonas	0.223	0	0.037	0.058	0.002	0	0.056
	Xylella	0.003	0	0.04	0.044	0.002	0.022	0.618
Spirochaetes	*Sphaerochaeta*	0.003	0.017	4.304	3.812	0.005	0	0.003
	Treponema	0.154	0.012	2.042	2.741	0.253	0.534	3.73
Synergistetes	HA73	0	0	0.029	0.023	0.381	0.047	0.023
	vadinCA02	0.009	0	0.143	0.099	0.014	0.034	0.073
	E6	0.018	0	0	0.003	0.025	0.047	0.01
Thermi	*Deinococcus*	0	0	0.011	0.006	0.155	0.019	0.01

Table S4 Commonly shared OTUs which were shared by at least 5 samples

Taxonomy		Relative abundance							Shared samples number
**Class level**	**Genus/Species level**	**CS-LXM**	**BJ-CZ**	**BJ-HX1**	**BJ-HX2**	**XM1**	**XM2**	**XM3**	
Actinobacteria	*Mycobacterium* sp.	0	0	0.001283	0.001365	0.000342	0.001117	0.000760	5
	Microbacterium sp.	0	0.000024	0.000998	0.001539	0.000023	0.000031	0	5
	Dietzia sp.	0	0	0.000171	0.000174	0.001687	0.000062	0.000099	5
	Microbacterium sp.	0	0	0.000371	0.000406	0.000410	0.000031	0.000033	5
	Propionicimonas sp.	0	0	0.000171	0.000174	0.000068	0.000310	0.000363	5
Alphaproteobacteria	*Rhodopseudomonas palustris*	0.000060	0.000024	0.000342	0.000552	0.099343	0.001427	0.002081	7
	Rhizobium selenitireducens	0.000030	0.000071	0.006161	0.007868	0.000752	0.000031	0.000396	7
	Brevundimonas diminuta	0.000332	0.000118	0.007729	0.003716	0.001755	0.000062	0.001321	7
	Unclassified	0	0.000142	0.040699	0.053133	0.004719	0.000248	0.002180	6
	Agrobacterium tumefaciens	0	0.000071	0.023273	0.029470	0.001938	0.000403	0.002247	6
	Mesorhizobium sp.	0	0.000024	0.000542	0.000552	0.011512	0.000093	0.000132	6
	Azospirillum sp.	0	0.000071	0.004506	0.003571	0.000935	0.000621	0.001553	6
	Devosia sp.	0	0.000047	0.000970	0.001219	0.005311	0.000186	0.000165	6
	Unclassified	0.000030	0	0.000086	0.000087	0.000023	0.002203	0.004592	6
	Unclassified	0.000091	0	0.000057	0.000116	0.005631	0.000186	0.000198	6
	Unclassified	0	0.000024	0.001027	0.001336	0.000205	0.000310	0.002346	6
	Methylosinus sp.	0.000060	0	0.000171	0.000058	0.002758	0.000124	0.000132	6
	Ochrobactrum sp.	0	0.000379	0.000428	0.000348	0.001664	0.000062	0.000033	6
	Unclassified	0.000030	0	0.000057	0.000029	0.001755	0.000062	0.000033	6
	Devosia sp.	0.000151	0	0.000200	0.000029	0.000205	0.000310	0.000496	6
	Hyphomicrobium sp.	0.000181	0	0.000086	0.000203	0.000091	0.000062	0.000165	6
	Sphingobium sp.	0	0.000308	0.000143	0.000145	0.000114	0.000031	0.000033	6
	Azospirillum lipoferum	0	0	0.005505	0.004616	0.026375	0.005429	0.004328	5
	Azospirillum zeae	0	0	0.001768	0.001074	0.000023	0.023672	0.013380	5
	Pleomorphomonas oryzae	0	0	0.000599	0.000871	0.000046	0.005802	0.004196	5
	Unclassified	0	0	0.001198	0.001161	0.000638	0.001334	0.001355	5
	Devosia sp.	0.000302	0	0.000941	0.000900	0.002941	0	0.000033	5
	Unclassified	0	0	0.000200	0.000116	0.004240	0.000248	0.000165	5

	Unclassified	0.000030	0	0.001968	0.002032	0.000023	0.000031	0	5
	Unclassified	0	0	0.000228	0.000261	0.002325	0.000217	0.000330	5
	Sphingopyxis sp.	0.000030	0	0	0.000087	0.000912	0.000900	0.001388	5
	Roseomonas sp.	0.000091	0	0.000086	0	0.000182	0.001272	0.001288	5
	Unclassified	0	0	0.000029	0.000029	0.002280	0.000310	0.000231	5
	Sphingobium sp.	0	0	0.000057	0.000116	0.001687	0.000279	0.000297	5
	Unclassified	0.000362	0	0.000057	0	0.000570	0.000124	0.000231	5
	Unclassified	0	0	0.000086	0.000087	0.000228	0.000465	0.000463	5
	Unclassified	0	0	0.000029	0.000058	0.000866	0.000186	0.000165	5
	Unclassified	0	0	0.000228	0.000319	0.000023	0.000124	0.000595	5
	Phaeospirillum sp.	0	0	0.000171	0.000174	0.000023	0.000186	0.000562	5
	Azospirillum sp.	0	0	0.000029	0.000029	0.000684	0.000124	0.000099	5
	Pleomorphomonas oryzae	0.000030	0.000024	0.000029	0	0	0.000310	0.000198	5
	Unclassified	0	0	0.000029	0.000232	0.000091	0.000124	0.000033	5
	Sphingomonas sp.	0.000060	0	0.000029	0.000029	0	0.000031	0.000231	5
Anaerolineae	WCHB1-05	0.000030	0	0.000428	0.000726	0.000319	0.008532	0.054643	6
	Unclassified	0.000543	0	0	0.000029	0.004331	0.000124	0.000198	5
	Unclassified	0.000211	0	0.000114	0.000029	0	0.000279	0.000463	5
Bacteroidia	*Dysgonomonas* sp.	0	0.000047	0.010268	0.006388	0.000023	0.002265	0.010803	6
	Dysgonomonas sp.	0	0.000024	0.001711	0.001161	0.000114	0.001893	0.000231	6
	Unclassified	0.000935	0	0.000143	0.000087	0.000068	0.000217	0.000562	6
	Unclassified	0.000030	0	0.000399	0.000755	0.000137	0.000062	0.000165	6
	Petrimonas sp.	0	0.000095	0.017540	0.015388	0	0.001551	0.004757	5
	Unclassified	0	0	0.000314	0.000261	0.002895	0.000062	0.000297	5
	Unclassified	0.000030	0	0.000086	0.000203	0.000023	0	0.000099	5
	Azospira oryzae	0.000091	0.055274	0.035166	0.030370	0.000046	0.000714	0.004790	7
	Acidovorax caeni	0.000634	0.000071	0.023615	0.023227	0.000091	0.004188	0.002379	7
	Alicycliphilus denitrificans	0.000151	0.000332	0.006560	0.008333	0.000023	0.000496	0.000099	7
	Comamonas testosteroni	0.000030	0.011728	0.008756	0.010714	0.000091	0	0.000033	6
	Achromobacter xylosoxidans	0	0.002890	0.002881	0.001916	0.000114	0.004933	0.006541	6
	Unclassified	0.001659	0.000047	0.003765	0.002323	0.000570	0.000124	0	6
	Hydrogenophaga sp.	0.000060	0	0.002196	0.001365	0.000205	0.000683	0.000892	6
	Unclassified	0.000030	0.000355	0.000941	0.000929	0	0.000031	0.000033	6

	Hydrogenophaga sp.	0.000121	0	0.000257	0.000261	0.000068	0.000186	0.000297	6
	Pandoraea sputorum	0	0.000024	0.001027	0.000987	0	0.011107	0.010638	5
	Xenophilus sp.	0	0.000024	0.001483	0.001481	0	0.000124	0.000033	5
	Unclassified	0	0	0.000428	0.000319	0.000547	0.000093	0.000099	5
	Azospira sp.	0	0.001232	0.000114	0.000058	0	0.000031	0.000033	5
Betaproteobacteria	*Acidovorax* sp.	0.000935	0.000142	0.000057	0.000087	0	0.000000	0.000132	5
	Unclassified	0	0.000024	0.000627	0.000319	0	0.000031	0.000099	5
	Unclassified	0	0.000284	0.000257	0.000232	0	0.000031	0.000066	5
	Unclassified	0	0.000284	0.000285	0.000174	0	0.000093	0.000033	5
	Achromobacter sp.	0	0	0.000114	0.000116	0.000023	0.000372	0.000198	5
	Azospira sp.	0	0.000474	0.000057	0.000174	0	0.000031	0.000033	5
	Unclassified	0	0.000190	0.000057	0.000145	0	0.000062	0.000033	5
	Unclassified	0	0	0.000086	0.000029	0.000023	0.000186	0.000132	5
	Unclassified	0	0.000047	0.000000	0.000029	0.000023	0.000093	0.000165	5
	Unclassified	0	0.000047	0.000029	0.000145	0	0.000062	0.000033	5
	Unclassified	0.000030	0	0.000029	0.000087	0	0.000062	0.000099	5
	Unclassified	0	0	0.000057	0.000029	0.000023	0.000031	0.000165	5
	Unclassified	0	0.000024	0.000114	0.000029	0	0.000031	0.000033	5
	Unclassified	0	0.000047	0	0.000029	0.000023	0.000031	0.000033	5
Clostridia	Unclassified	0.000483	0	0.000456	0.000697	0.000091	0.000031	0.000165	6
	Unclassified	0	0	0.003537	0.003107	0.000228	0.000465	0.000793	5
	Unclassified	0.000030	0	0.000941	0.001016	0.000000	0.000062	0.000132	5
	Unclassified	0	0	0.000029	0.000116	0.000023	0.000062	0.000066	5
Deltaproteobacteria	*Geobacter sulfurreducens*	0	0.000261	0.057413	0.022850	0.000479	0.000558	0.000165	6
	Geobacter sp.	0	0.000024	0.000542	0.000581	0	0.009245	0.037431	5
	Unclassified	0.000030	0	0.000143	0.000145	0	0.000993	0.000859	5
	Unclassified	0	0	0.000342	0.000465	0.000274	0.000031	0.000132	5
Flavobacteriia	*Kaistella* sp.	0	0	0.000228	0.000116	0.000068	0.000434	0.000132	5
Gammaproteobacteri[illegible]	*Stenotrophomonas acidaminip[illegible]*	0.000030	0.000900	0.002538	0.003629	0.001322	0.000310	0.001388	7
	Acinetobacter sp.	0.000030	0	0.000057	0.000087	0.000091	0.000186	0.000396	6
	Acinetobacter guillouiae	0.000332	0.078705	0.020164	0.011759	0.000023	0	0	5
	Acinetobacter gerneri	0.000030	0.000095	0.022104	0.009175	0.000023	0	0	5
	Pseudomonas sp.	0.000151	0.007771	0.000200	0	0	0.000124	0.000132	5

Gammaproteobacteria	Unclassified	0	0.001161	0.000057	0.000029	0	0.000776	0.004559	5
	Unclassified	0.000030	0.002654	0.000029	0	0	0.000031	0.000033	5
	Pseudomonas veronii	0.000815	0.000024	0.000485	0.000116	0.000023	0	0	5
	Unclassified	0	0	0.000542	0.000581	0.000046	0.000093	0.000033	5
	Unclassified	0	0.000047	0.000029	0.000145	0.000023	0	0.000033	5
Ignavibacteria	Unclassified	0	0.000166	0.032029	0.033941	0.000160	0.000776	0.000760	6
[Lentisphaeria]	Unclassified	0	0	0.000143	0.000406	0.000023	0.000031	0.000165	5
	Unclassified	0.000030	0	0	0.000029	0.000091	0.000093	0.000066	5
Planctomycetia	Unclassified	0.000030	0	0	0.000058	0.022727	0.000186	0.000496	5
	Unclassified	0	0	0.000086	0.000029	0.000068	0.001644	0.000496	5
Solibacteres	Candidatus *Solibacter* sp.	0	0.000024	0.000314	0.000145	0	0.000403	0.001255	5
Sphingobacteriia	Unclassified	0.003922	0.000071	0	0	0.000068	0.000279	0.000396	5
	Sphingobacterium mizutaii	0.000030	0.000047	0	0.000029	0	0.000093	0.000033	5
Spirochaetes	*Treponema* sp.	0.000030	0.000047	0.015002	0.020731	0.000068	0.000683	0.028676	7
	Treponema sp.	0.000030	0.000024	0.001740	0.001974	0.000251	0.000683	0.000727	7
	Treponema sp.	0	0	0.000257	0.000232	0.000023	0.001365	0.000727	5
Synergistia	vadinCA02	0.000030	0	0.001084	0.000755	0	0.000186	0.000363	5
TM7-3	Unclassified	0	0	0.000257	0.000290	0.000205	0.000155	0.000231	5
Unclassified	Unclassified	0	0	0.000171	0.000232	0.000707	0.000062	0.000066	5
Unclassified	Unclassified	0	0	0.000399	0.000232	0.000433	0.000031	0.000099	5
Unclassified	Unclassified	0	0.000047	0.000029	0.000029	0	0.000031	0.000033	5

Table S5 OTUs with an average relative abundance higher than 0.1%

OTU ID	Taxonomy		Relative abundance							Shared samples number
	Class level	Genus/Species level	CS-LXM	BJ-CZ	BJ-HX1	BJ-HX2	XM1	XM2	XM3	
36744	Alphaproteobacteria	*Stappia indica*	0	0	0	0.000406	0.200100	0.000434	0.000165	4
28339		*Agrobacterium tumefaciens*	0	0.000071	0.023273	0.029470	0.001938	0.000403	0.002247	6
14215		*Azospirillum* sp.	0	0.000071	0.004506	0.003571	0.000935	0.000621	0.001553	6
16867		*Azospirillum lipoferum*	0	0	0.005505	0.004616	0.026375	0.005429	0.004328	5
32034		*Azospirillum zeae*	0	0	0.001768	0.001074	0.000023	0.023672	0.013380	5
16433		*Brevundimonas diminuta*	0.000332	0.000118	0.007729	0.003716	0.001755	0.000062	0.001321	7
6815		*Devosia* sp.	0	0.000047	0.000970	0.001219	0.005311	0.000186	0.000165	6
23816		*Hyphomicrobium aestuarii*	0	0	0	0.000058	0.027651	0.001582	0.000694	4
24361		*Mesorhizobium* sp.	0	0.000024	0.000542	0.000552	0.011512	0.000093	0.000132	6
32126		*Pleomorphomonas oryzae*	0	0	0.000599	0.000871	0.000046	0.005802	0.004196	5
8834		*Rhizobiales* sp.	0	0	0	0	0.010646	0	0	1
31858		*Rhizobiales* sp.	0	0	0.004107	0.004297	0.000023	0	0.000826	4
27905		*Rhizobium selenitireducens*	0.000030	0.000071	0.006161	0.007868	0.000752	0.000031	0.000396	7
33927		*Rhodopseudomonas* sp.	0	0	0	0.000029	0.016390	0.000093	0.000033	4
29034		*Rhodopseudomonas palustris*	0.000060	0.000024	0.000342	0.000552	0.099343	0.001427	0.002081	7
27012		Unclassified	0.000030	0	0.000086	0.000087	0.000023	0.002203	0.004592	6
6374		Unclassified	0	0.000142	0.040699	0.053133	0.004719	0.000248	0.002180	6
2284	Anaerolineae	WCHB1-05 sp.	0.000030	0	0.000428	0.000726	0.000319	0.008532	0.054643	6
14244		*Anaerolinea* sp.	0	0	0	0	0.024095	0.000155	0.000033	3
29046		Unclassified	0.011978	0	0	0	0.000046	0	0	2
3471	Bacilli	*Sporosarcina* sp.	0	0.000047	0.004078	0.006765	0.000023	0	0	4
29399		*Bacillus* sp.	0	0.007108	0	0	0	0	0	1
22405	Bacteroidia	*Bacteroides* sp.	0	0.000047	0	0	0.000091	0.081472	0.034920	4
236		Unclassified	0	0	0	0	0.000068	0.070272	0.041726	3
4653		*Petrimonas* sp.	0	0.000095	0.017540	0.015388	0	0.001551	0.004757	5
8154		*Dysgonomonas* sp.	0	0.000047	0.010268	0.006388	0.000023	0.002265	0.010803	6
9625		*Parabacteroides* sp.	0.003077	0.008624	0	0	0	0.008842	0.004658	4
12857		*Bacteroides* sp.	0.000030	0	0	0	0	0.006794	0.003238	3
33416		Unclassified	0	0	0	0	0.007317	0.001520	0.001057	3
33362		Unclassified	0.001297	0	0	0	0.007568	0.000248	0.000429	4
24023		Unclassified	0.000030	0	0.000057	0	0	0.001117	0.007136	4

341		*Bacteroides* sp.	0	0	0	0	0	0.002978	0.004923	2
15238		Unclassified	0	0	0.003109	0.004471	0	0	0	2
15694		*Bacteroidales* sp.	0	0	0.003879	0.003397	0	0	0	2
15613	Betaproteobacteria	*Azospira oryzae*	0.000091	0.055274	0.035166	0.030370	0.000046	0.000714	0.004790	7
19577		*Azospira* sp.	0	0.000142	0.031002	0.030893	0	0	0.000165	4
14184		*Acidovorax caeni*	0.000634	0.000071	0.023615	0.023227	0.000091	0.004188	0.002379	7
36728		*Comamonas testosteroni*	0.000030	0.011728	0.008756	0.010714	0.000091	0	0.000033	6
12692		*Rubrivivax gelatinosus*	0.005431	0	0	0	0	0.002048	0.023060	3
4988		*Pandoraea sputorum*	0	0.000024	0.001027	0.000987	0	0.011107	0.010638	5
14908		*Achromobacter xylosoxidans*	0	0.002890	0.002881	0.001916	0.000114	0.004933	0.006541	6
14376		*Dechloromonas* sp.	0.018646	0.000024	0	0	0	0	0	2
3002		Unclassified	0.017107	0.000024	0	0	0.000046	0	0	3
18877		*Alicycliphilus denitrificans*	0.000151	0.000332	0.006560	0.008333	0.000023	0.000496	0.000099	7
26240		*Acidovorax* sp.	0	0.000024	0.006046	0.009291	0	0	0	3
28507		*Comamonas koreensis*	0	0.000166	0.006075	0.008246	0	0	0	3
8548		Candidatus *Accumulibacter* sp.	0.010862	0.000047	0	0	0	0	0	2
27536		Unclassified	0.000060	0.009951	0.000143	0.000174	0	0	0	4
3404		Unclassified	0	0.001303	0	0	0.000775	0.001458	0.006773	4
31026		*Cupriavidus* sp.	0	0	0.006218	0.003571	0	0	0	2
7574		Unclassified	0.007392	0	0	0	0.000957	0.000248	0.000198	4
13251		Unclassified	0.001659	0.000047	0.003765	0.002323	0.000570	0.000124	0	6
35417		KD1-23	0.007905	0.000024	0	0	0	0	0	2
726		Candidatus *Accumulibacter* sp.	0.007664	0	0	0	0	0	0	1
3195		Unclassified	0	0	0.002339	0.003803	0	0.000558	0.000859	4
23951	Clostridia	Unclassified	0	0	0	0	0.000023	0.024820	0.008854	3
35699		Unclassified	0	0	0	0	0.000023	0.003103	0.028181	3
8022		*Oscillospira* sp.	0	0	0	0	0.000023	0.027023	0.002544	3
6482		Unclassified	0	0	0	0	0.000114	0.017436	0.004790	3
14484		Unclassified	0	0	0	0	0.000023	0.003071	0.012951	3
34970		*Clostridium* sp.	0	0	0	0	0.000023	0.014861	0.000429	3
5971		*Clostridium* sp.	0	0	0	0	0	0.014427	0.000496	2
25953		Unclassified	0	0	0	0	0	0.009773	0.004262	2
12871		*Caloramator* sp.	0	0	0	0	0	0.007167	0.006442	2
28159		Unclassified	0	0	0.002339	0.001626	0	0.003537	0.003469	4
24890		*Oscillospira* sp.	0	0	0	0	0.000023	0.009370	0.000694	3

2879		*Thermosinus* sp.	0	0	0	0	0.000023	0.006112	0.002015	3
29464		*Clostridia* sp.	0	0	0.003537	0.003107	0.000228	0.000465	0.000793	5
960		*Geobacter sulfurreducens*	0	0.000261	0.057413	0.022850	0.000479	0.000558	0.000165	6
14436		*Geobacter* sp.	0	0.000024	0.000542	0.000581	0	0.009245	0.037431	5
30154	Deltaproteobacteria	Unclassified	0	0.000118	0.011380	0.021747	0.000046	0	0	4
2783		*Desulfovibrio* sp.	0	0	0	0.000029	0	0.006081	0.019162	3
5002		Unclassified	0	0.000024	0.009041	0.005081	0	0	0	3
3891		*Desulfovibrio* sp.	0	0	0	0	0	0.006019	0.001817	2
4350	Epsilonproteobacteria	*Arcobacter* sp.	0.062304	0	0	0	0	0	0	1
10884		*Flavobacterium* sp.	0	0	0.012635	0.007491	0	0	0	2
30066	Flavobacteriia	Unclassified	0	0	0	0	0.000137	0.000248	0.011431	3
32502		*Chryseobacterium* sp.	0.000060	0.011538	0	0	0	0	0	2
2032		*Chryseobacterium* sp.	0	0	0	0.000029	0	0.004871	0.004130	3
161		*Pseudomonas aeruginosa*	0.001448	0.545252	0.000257	0	0	0	0.000033	4
4293		*Acinetobacter guillouiae*	0.000332	0.078705	0.020164	0.011759	0.000023	0	0	5
15511		*Xanthomonas* sp.	0	0	0.000713	0.001132	0	0.000155	0.047673	4
9562		*Acinetobacter gerneri*	0.000030	0.000095	0.022104	0.009175	0.000023	0	0	5
35094		*Acinetobacter* sp.	0.000030	0.000142	0.015059	0.010423	0	0	0	4
7874		*Acinetobacter guillouiae*	0.000091	0.023053	0.000086	0.000029	0	0	0	4
21445		*Pseudomonas pseudoalcaligene*	0	0.000024	0.014916	0.008188	0.000023	0	0	4
19119	Gammaproteobacteria	*Acinetobacter soli*	0	0.000071	0.013719	0.006417	0	0	0	3
20847		*Pseudomonas* sp.	0.000091	0.017959	0	0	0	0	0	2
1081		*Pseudomonas aeruginosa*	0.000060	0.014547	0	0	0	0	0.000033	3
15954		*Dokdonella* sp.	0	0	0.005333	0.005400	0	0	0	2
19287		*Stenotrophomonas acidaminiph*	0.000030	0.000900	0.002538	0.003629	0.001322	0.000310	0.001388	7
14064		Unclassified	0.000091	0.008411	0.000029	0	0	0	0	3
8490		*Pseudomonas* sp.	0.000151	0.007771	0.000200	0	0	0.000124	0.000132	5
21182		*Acinetobacter* sp.	0	0	0.005562	0.002439	0	0	0	2
23747	Holophagae	Unclassified	0	0	0	0	0	0.008625	0.097691	2
13421	Ignavibacteria	Unclassified	0	0.000166	0.032029	0.033941	0.000160	0.000776	0.000760	6
29337	[Lentisphaeria]	*Victivallis* sp.	0	0.000024	0.018738	0.040909	0.000023	0	0	4
15842	Nitrospira	*Nitrospira* sp.	0.014090	0.000024	0	0	0	0	0	2
28729	OPB56	Unclassified	0	0	0	0	0.000980	0.020725	0.009118	3
522		*Gemmata* sp.	0	0	0	0.000029	0.042970	0.000714	0.000099	4
1562	Planctomycetia	Unclassified	0.000030	0	0	0.000058	0.022727	0.000186	0.000496	5

34450	Planctomycetia	*Planctomyces* sp.	0	0	0	0	0.007523	0.000062	0.000033	3
8714		*Planctomyces* sp.	0	0	0	0	0.007431	0.000062	0	2
3637	Sphingobacteriia	*Pedobacter* sp.	0.000030	0.008908	0	0	0	0	0	2
34047		Unclassified	0.007784	0.000024	0	0	0	0	0	2
35301	Spirochaetes	*Treponema* sp.	0.000030	0.000047	0.015002	0.020731	0.000068	0.000683	0.028676	7
34368		Sphaerochaeta sp.	0	0.000118	0.032400	0.027989	0.000046	0	0	4
21943	Unclassified	Unclassified	0	0.000071	0.003907	0.005865	0.000023	0	0	4
